# Treatment patterns of targeted and nontargeted therapies and survival effects in patients with locally advanced head and neck cancer in Taiwan

**DOI:** 10.1186/s12885-023-11061-4

**Published:** 2023-06-20

**Authors:** Szu-Han Hu, Ming-Yii Huang, Chung-Yu Chen, Hui-Min Hsieh

**Affiliations:** 1 Master Program in Clinical Pharmacy, School of Pharmacy, Kaohsiung Medical University, Kaohsiung, Taiwan; 2 Department of Radiation Oncology, Kaohsiung Medical University Hospital, Kaohsiung Medical University, Kaohsiung, Taiwan; 3 Department of Pharmacy, Kaohsiung Medical University Hospital, Kaohsiung, Taiwan; 4 Department of Medical Research, Kaohsiung Medical University Hospital, Kaohsiung, Taiwan; 5 Center for Big Data Research, Kaohsiung Medical University, Kaohsiung, Taiwan; 6 Department of Public Health, Department of Medical Research, Department of Community Medicine, Research Center for Precision Environmental Medicine, Kaohsiung Medical University Hospital, 100 Shih-Chung 1st Road, San-Ming Dist, Kaohsiung, 807 Taiwan

**Keywords:** Head and Neck Neoplasms, Molecular targeted therapy, Cetuximab, Chemoradiotherapy

## Abstract

**Background:**

Taiwan’s National Health Insurance has covered targeted therapy, namely cetuximab, for locally advanced head and neck cancers (LAHNC) since July 2009. This study examines treatment trends and survival effects of locally advanced head and neck cancer patients before and after Taiwan’s National Health Insurance covered cetuximab.

**Methods:**

We examined treatment trends and survival effects for patients with LAHNC using Taiwan’s National Health Insurance Research Database. Patients who received treatment within 6 months were categorized as either nontargeted or targeted therapy groups. We analyzed treatment trends with the Cochran-Armitage trend test and explored factors associated with treatment selection and survival effects using multivariable logistic regression and Cox proportional hazards models.

**Results:**

Of the 20,900 LAHNC patients included in the study, 19,696 received nontargeted therapy, while 1,204 received targeted therapy. Older patients with more comorbid conditions, advanced stages and patients with hypopharynx and oropharynx cancers were more likely to receive targeted therapy with concomitant cetuximab treatment. Patients who received targeted therapy in addition to other treatment modalities had a greater risk of one-year and long-term all-cause mortality or cancer-specific mortality than those without receiving targeted therapy (P < 0.001).

**Conclusions:**

Our study found an increasing trend in cetuximab utilization among LAHNC after reimbursement in Taiwan, but overall usage rates were low. LAHNC patients receiving cetuximab with other treatments had higher mortality risk than those receiving cisplatin, suggesting cisplatin may be preferred. Further research is needed to identify subgroups that could benefit from concomitant cetuximab treatment.

**Supplementary Information:**

The online version contains supplementary material available at 10.1186/s12885-023-11061-4.

## Introduction

Head and neck cancers (HNC) are malignant tumors in the upper aerodigestive tract, including the oral cavity, pharynx, and larynx. The most common histological type is squamous cell carcinoma, which accounts for more than 90% of cases [1, 2]. Nearly 900,000 new incident HNC cases and almost 507,000 deaths occur each year, with 40–50% 5-year mortality worldwide [2]. HNCs are the sixth most common cancer in Taiwan, with an annual age-standardized incidence rate of 22.1 per 100,000 people and an annual mortality rate of 8.9 per 100,000 people. HNC incidence and prevalence is much higher in men than in women, and is the fourth most common cancer in men [3].

HNCs usually present as advanced disease due to a paucity of specific symptoms. Over 50% of HNC patients are newly diagnosed at a locally advanced stage in Taiwan[3]. Patients with locally advanced HNC (LAHNC) often have a poor prognosis, with average 1-year and 5-year survival rates of approximately 80% and 56%, respectively [3]. Treatment patterns may affect antitumor outcomes as well as survival rates. According to the National Comprehensive Cancer Network (NCCN) guidelines, patients with LAHNC usually receive a combination of treatments, including surgery, radiotherapy, chemotherapy, or targeted therapy [1, 4, 5]. Surgery is more often performed for oral cavity cancer. Oropharynx, hypopharynx, and larynx cancer patients usually receive concurrent systemic therapy with radiotherapy in a definitive setting for organ and functional preservation [1, 4, 5].

The most preferred regimen of systemic therapy is high-dose cisplatin. Cisplatin is an alkylating agent that can be used as cytotoxic chemotherapy. Concurrent chemotherapy increases acute toxicity due to its cytotoxicity, including nephrotoxicity and ototoxicity; patients receiving cisplatin experience more adverse reactions including nausea, vomiting, acute kidney injury, or tinnitus[6]. Cetuximab is an alternative targeted therapy that binds to the epidermal growth factor receptor to inhibit cell proliferation, and may be used as an option for patients who are ineligible for cytotoxic chemotherapy based on certain criteria[7–11]. Since July 2009, Taiwan’s National Health Insurance has covered cetuximab (Erbitux®) for locally advanced oropharynx, hypopharynx, and larynx cancers with some limiting conditions, including one of the following criteria: age ≥ 70 years, creatinine clearance rate < 50 mL/min, hearing loss (over 25 dB of average hearing loss at 500, 1,000, 2,000 Hz), or resistance to platinum-based chemotherapy. The maximum total treatment course is 8 infusions with pre-reviewed [12].

Several empirical studies have investigated treatment trends for HNC patients in the United States and European Counties. For example, Baxi et al. (2016) examined treatment trends in chemoradiotherapy in elderly patients using the Surveillance, Epidemiology, and End Results (SEER) Medicare database from 2001 to 2009 in the United States, spanning 2006, the year cetuximab was approved for HNCs [13]. They found that the proportion of patients undergoing surgery or radiation alone decreased, but the rate of chemoradiotherapy use increased. Findings were similar in Schlichting et al. (2019) [14]. Hermanns et al. (2021) used nationwide diagnosis-related-group inpatient data in Germany to examine treatment rates and trends from 2005 to 2018 and found that use of surgery or radiotherapy alone decreased, and use of chemotherapy and biologics increased by the year [15]. However, very few studies have been conducted in Asian countries. Only one study, by Yamada et al. (2019), examined treatment changes from 2008 to 2015 in Japan, and found that the cisplatin-based regime was most often used as chemotherapy [16].

Moreover, mixed clinical outcomes of concurrent radiotherapy with cetuximab (targeted therapy) versus cisplatin (non-targeted therapy) in LAHNC were found in existing literature. For example, Xiang et al. utilized the SEER-Medicare database to perform a population-based cohort study from 2004 to 2013 in the US. The hazard ratio (HR) of all-cause mortality for concurrent radiotherapy with cetuximab was 1.57 [1.30–1.90] (*p*-value < 0.001) [17]. Tang et al. conducted a meta-analysis evaluating the efficacy of cetuximab versus cisplatin in the locally advanced head and neck squamous cell carcinoma, and found patients treated cetuximab had worse overall survival (HR = 1.96, 95%CI, 1.56–2.44, P < 0.001) and progression-free survival (HR = 2.70, 95%CI, 1.67–3.45, P < 0.001)[18]. However, Hu et al.(2014) conducted a retrospective single-center LAHNC cohort, and compared cetuximab with concurrent radiotherapy (BioRT) to split-dose cisplatin-based concurrent chemoradiotherapy (SDCCRT) from 2009 to 2012, and found non-significant difference in 3-year overall survival (P = 0.879) as the result of small patient sample size (n = 170) [19]. Lu et al. (2018) conducted a single center cohort (n = 23) in the U.S. and found non-significant difference in 2-year overall survival between two regimens[20]. Therefore, further studies are needed to investigate the comparative effectiveness between two regimens among patients with LAHNC.

Given that financial reimbursement and clinical practice differ in different health systems, it is important to increase knowledge regarding treatment trends with new substitute targeted therapy and regarding treatment patterns and associated health outcomes in different ethnicities as well as in health systems. This study aimed to estimate the treatment trends in first-course treatment in LAHNC patients before and after cetuximab became reimbursable in Taiwan, and to evaluate treatment selection factors and survival effects between LAHNC patients who did and did not receive targeted therapies. Specially, we used population-based Taiwan’s National Health Insurance Research Database and Taiwan Cancer Registry to analyze treatment patterns from 2008 to 2016, spanning the year 2009, for the first targeted therapy (cetuximab) to be reimbursed, and compared one-year and long-term mortality by treatment patterns with and without targeted therapy.

## Methods

### Study design and data source

Using the Taiwan National Health Insurance Research Database (NHIRD), the Taiwan Cancer Registry (TCR), and the Taiwan Death Registry (TDR), we conducted a retrospective study on a nationwide population. The NHIRD includes data on more than 99% of Taiwan’s population, including birth year, sex, monthly payroll, and comorbidities such as disease diagnoses and outpatient and inpatient care [21, 22]. The TCR contains records of cancer diagnoses and dates, dating back to 1979 and up to 2016 [3]. The TDR, from 1971 to 2019, contains information on accurate causes of death and dates for all residents of Taiwan [21]. All data were analyzed in 2021–2022 at the Health and Welfare Data Science Center of the Ministry of Health and Welfare, a national data warehouse operated by the Taiwanese government. The study followed the Helsinki Declaration of the World Medical Association and was approved by the Institutional Review Board of Kaohsiung Medical University (KMUHIRB-E(II)-20,180,301). Since all three population-based datasets were encrypted and de-identified to protect patient privacy, informed consent from patients was waived.

### Study population

**Fig. 1:**
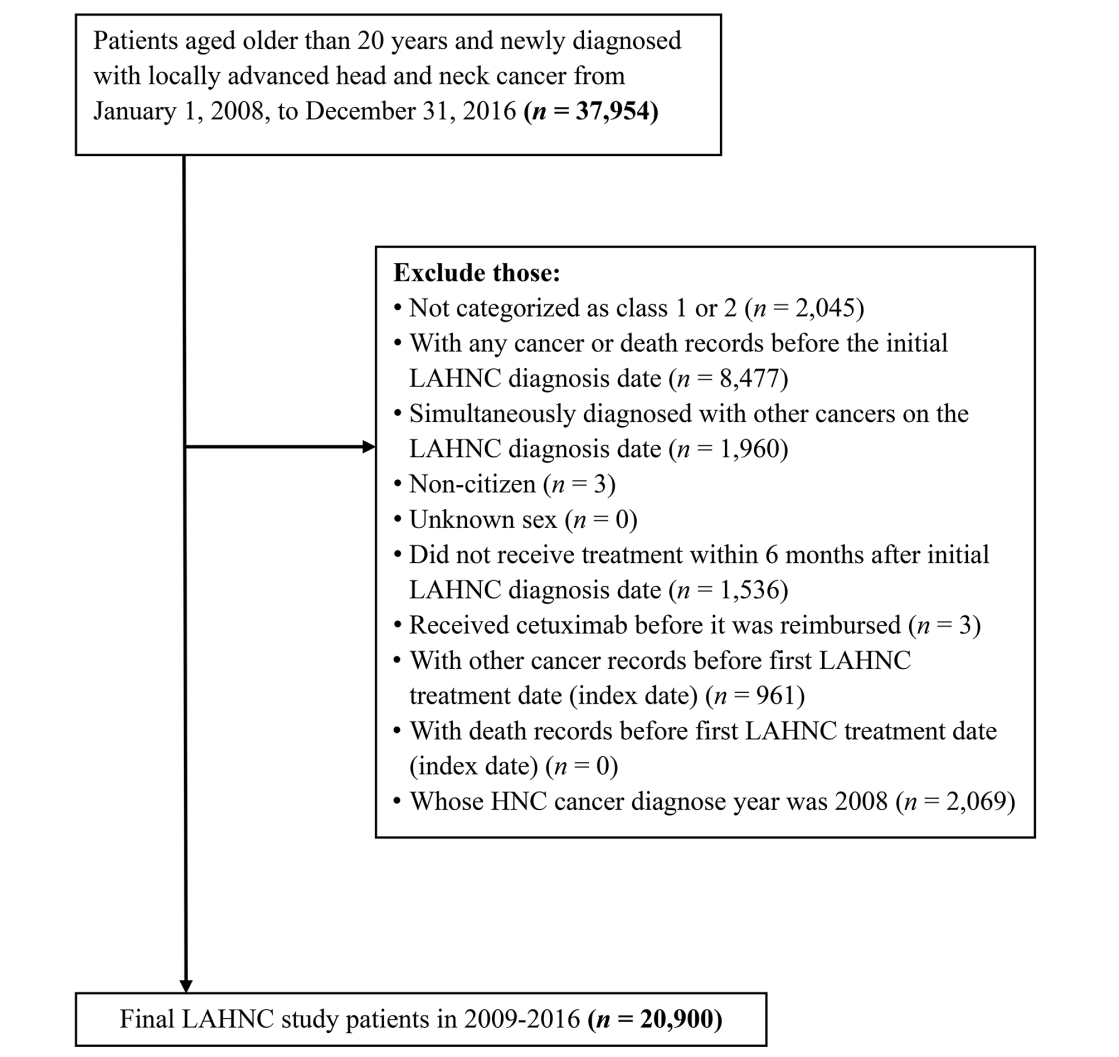
Inclusion and exclusion criteria of the study

Patients aged older than 20 years, categorized as class 1 or 2 (receiving all or part of the first-course treatments in the hospitals reporting cancer registry information), newly diagnosed with LAHNC (ICD-O-3: C00-C10, 12–14, 32; ICD-O-FT: 140–146, 148–149, 161) between 2008 and 2016 in the TCR long-form database, and receiving first-course treatments within 6 months after the initial diagnosis date were included. From 2008 to 2016, 37,954 patients aged older than 20 years had a new LAHNC diagnosis record. Of these, 14,014 were excluded because they were not categorized as class 1 or 2 (*n* = 2,045), had any cancer or death record before the initial LAHNC diagnosis date (*n* = 8,477), were simultaneously diagnosed with other cancers on the LAHNC diagnosis date of LAHC (*n* = 1,960), were non-citizen (*n* = 3), did not receive treatment within 180 days of the initial LAHNC diagnosis date (*n* = 1 536), or received cetuximab before it became reimbursable (*n* = 3); 961 patients with other cancer records before the first LAHNC treatment date were also excluded. A total of 22,969 patients from 2008 to 2016 were analyzed for trends in treatment modalities. Given that targeted therapy was reimbursed starting in 2009, we included LAHNC patients (*n* = 20 900) from 2009 to 2016 in the analysis, categorized as nontargeted and targeted therapy groups according to whether they received targeted therapy, to evaluate treatment modalities and associated survival effects. Figure 1 provides detailed inclusion and exclusion criteria for the study sample.

### Measurements

The outcomes of interest include treatment patterns, trends in 2008–2016, selection factors, and survival effects. All the treatment combinations of surgery, radiotherapy, chemotherapy, and targeted therapy were analyzed. The modalities of systemic therapy agents used in four conditions were calculated separately, including induction therapy, definitive radiochemotherapy, postoperative radiochemotherapy, and others. We regarded the first course of treatment as surgery, radiotherapy, chemotherapy, or targeted therapy given for LAHNC within 6 months after the initial diagnosis date. The commonly used chemotherapy agents for LAHNC were identified, including cisplatin, carboplatin, fluorouracil (5-FU), uracil-tegafur, paclitaxel, docetaxel, cyclophosphamide, ifosfamide, methotrexate, vincristine, doxorubicin, epirubicin, bleomycin, hydroxyurea; the targeted therapy agent, cetuximab, was also identified. The first date patients received these treatments was defined as the index date. Sex, age, primary tumor site, cancer stage, histology, smoking history, betel nut chewing history, and drinking history were extracted. Charlson comorbidity index was calculated to evaluate comorbid conditions [23, 24]. The frequency of systemic therapy was shown as prescribed times. All patients were followed until the first of death or December 31, 2019. One-year and long-term mortality were estimated. A death due to HNC was regarded as cancer-specific mortality; a death due to any reason was considered all-cause mortality. Coding algorithms for the measurements are listed in Supplementary eTables 1 to 5.

### Statistical analyses

Baseline characteristics were analyzed using descriptive statistics. Mean and standard deviation (SD) are presented for continuous variables. Student’s t-test or Fisher’s exact test was performed to compare the difference between nontargeted and targeted therapy groups. For categorical variables, number and proportion (%) are presented, and the chi-square (χ^2^) test was performed. Treatment trends from 2008 to 2016 were estimated using the Cochran-Armitage trend test. Multivariable logistic regressions were performed to evaluate the odds ratio (OR) and 95% confidence interval (CI) for factors associated with targeted therapy use. Given the concern that maximum likelihood estimates of the conventional logistic regressions may be biased by small numbers of events over a large sample size, the Firth penalized likelihood estimation model was used to reduce bias in generalized linear models [25]. Covariates include age category, sex, cancer stage, tumor site, CCI score category, surgery, radiotherapy, chemotherapy, drinking history, betel nut chewing history, smoking history, and diagnosis year. Total follow-up time, mortality (deaths/100,000 person-years), and mortality rate ratios were also calculated. Assessing the risk of death between nontargeted and targeted therapy groups, we used multivariate Cox proportional hazard models to calculate the hazard ratio (HR) and 95% CI. Age category, sex, cancer stage, tumor site, CCI score category, treatment modality, betel nut chewing history, smoking history, and diagnosis year were adjusted for. A *P* from the two-tailed test less than 0.05 was considered statistically significant for statistical testing. All analyses were performed using SAS software, version 9.4 (SAS Institute Inc., Cary, NC).

## Results

**Fig. 2:**
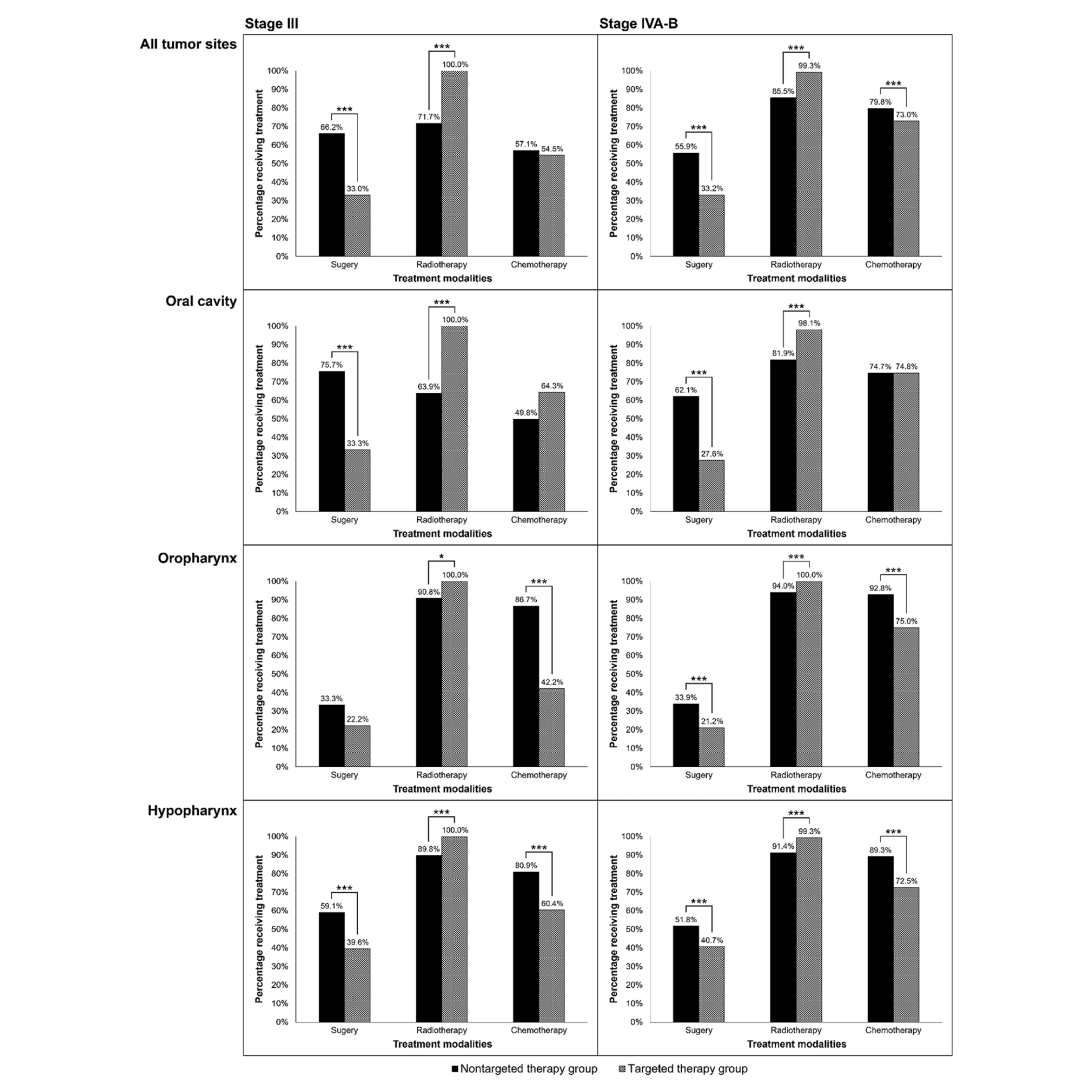
Comparisons of treatment modalities between LAHNC patients who did and did not receive targeted therapy by cancer stage and tumor site

Table 1 shows treatment modalities and treatment trends from 2008 to 2016. About 57%, 83%, and 75% of patients underwent surgery, radiotherapy, and chemotherapy, respectively, each year. The proportion undergoing surgery decreased over time (*P* for Cochran-Armitage trend test < 0.001). In 2009, 68 (3.03%) patients received targeted therapy; this number increased during the following years. In the nontargeted therapy group, almost all (99.42%) patients also received radiotherapy, with a stable proportion receiving chemotherapy each year. Figure 2 provides results of comparisons of treatment modalities between LAHNC patients who did and not receive targeted therapy by cancer stage and tumor site. Patients who received targeted therapy tended to also receive radiotherapy and were less likely to undergo surgery, while chemotherapy use did not significantly differ in stage III cancer for all tumor sites. Supplementary eTables 6, 7, 8, 9, 10 and 11 provide further information regarding modalities and the top five systemic therapies (induction therapy, definitive radiochemotherapy, postoperative radiochemotherapy, and others) of first-course treatment within 6 months in the nontargeted and targeted therapy groups.

**Table 1:** Treatment modality trends between locally advanced head and neck cancer patients who did and did not receive target therapy in 2008–2016

Treatment modalities	All	2008	2009	2010	2011	2012	2013	2014	2015	2016	*P*
Overall	22,969	2,069	2,243	2,399	2,522	2,480	2,735	2,823	2,761	2,937	
Any surgery	13,109 (57.07%)	1,281 (61.91%)	1,360 (60.63%)	1,425 (59.4%)	1,482 (58.76%)	1,404 (56.61%)	1,515 (55.39%)	1,566 (55.47%)	1,541 (55.81%)	1,535 (52.26%)	< 0.001
Any radiotherapy	19,161 (83.42%)	1,693 (81.83%)	1,807 (80.56%)	1,980 (82.53%)	2,078 (82.39%)	2,058 (82.98%)	2,324 (84.97%)	2,398 (84.95%)	2,340 (84.75%)	2,483 (84.54%)	< 0.001
Any chemotherapy	17,150 (74.67%)	1,546 (74.72%)	1,582 (70.53%)	1,773 (73.91%)	1,891 (74.98%)	1,846 (74.44%)	2,062 (75.39%)	2,169 (76.83%)	2,084 (75.48%)	2,197 (74.8%)	0.002
Any targeted therapy	1,204 (5.24%)	0 (0%)	68 (3.03%)	142 (5.92%)	161 (6.38%)	158 (6.37%)	161 (5.89%)	146 (5.17%)	174 (6.3%)	194 (6.61%)	< 0.001
Without targeted therapy group	21,765	2,069	2,175	2,257	2,361	2,322	2,574	2,677	2,587	2,743	
Any surgery	12,710 (58.4%)	1,281 (61.91%)	1,333 (61.29%)	1,368 (60.61%)	1,430 (60.57%)	1,353 (58.27%)	1,455 (56.53%)	1,525 (56.97%)	1,475 (57.02%)	1,490 (54.32%)	< 0.001
Any radiotherapy	17,964 (82.54%)	1,693 (81.83%)	1,739 (79.95%)	1,838 (81.44%)	1,917 (81.19%)	1,900 (81.83%)	2,163 (84.03%)	2,252 (84.12%)	2,169 (83.84%)	2,293 (83.59%)	< 0.001
Any chemotherapy	16,313 (74.95%)	1,546 (74.72%)	1,535 (70.57%)	1,674 (74.17%)	1,776 (75.22%)	1,731 (74.55%)	1,957 (76.03%)	2,068 (77.25%)	1,960 (75.76%)	2,066 (75.32%)	< 0.001
With targeted therapy group	1,204	0	68	142	161	158	161	146	174	194	
Any surgery	399 (33.14%)	0 (%)	27 (39.71%)	57 (40.14%)	52 (32.3%)	51 (32.28%)	60 (37.27%)	41 (28.08%)	66 (37.93%)	45 (23.2%)	0.010
Any radiotherapy	1,197 (99.42%)	0 (%)	68 (100%)	142 (100%)	161 (100%)	158 (100%)	161 (100%)	146 (100%)	171 (98.28%)	190 (97.94%)	0.001
Any chemotherapy	837 (69.52%)	0 (%)	47 (69.12%)	99 (69.72%)	115 (71.43%)	115 (72.78%)	105 (65.22%)	101 (69.18%)	124 (71.26%)	131 (67.53%)	0.624

Note: p-values were generated using the Cochran–Armitage test for trend analysis

Table 2 shows the baseline characteristics of the study subjects. Among 20,900 study patients, 19,696 did not and 1,204 did receive targeted therapy within 180 days. The mean age among the overall population was 55.33 years, and the targeted therapy group was older than the nontargeted therapy group (64.95 versus 54.72). Most subjects were male (92.98%), in stage IVA-IVB (79.00%), with the histological type squamous cell carcinoma (96.89%). The CCI was higher for the targeted therapy group (1.83 ± 2.19 versus 1.00 ± 1.61). All baseline characteristics showed significant differences between these two groups.

**Table 2:** Baseline demographic and clinical characteristics between locally advanced head and neck cancer patients who did and did not receive targeted therapy in 2009–2016

Characteristics	Overall	W/t targeted therapy group	With targeted therapy group	*P*
N	20,900	19,696	1,204	
Age (years) (Mean, SD)	55.33 ± 11.22	54.72 ± 10.88	64.95 ± 12.02	< 0.001
Age category (N, %)				
≤ 44 years	3,352 (16.04%)	3,305 (16.78%)	47 (3.9%)	< 0.001
45–54 years	7,274 (34.8%)	7,052 (35.8%)	222 (18.44%)	
55–64 years	6,357 (30.42%)	6,047 (30.7%)	310 (25.75%)	
≥ 65 years	3,917 (18.74%)	3,292 (16.71%)	625 (51.91%)	
Sex (N, %)				
Male	19,433 (92.98%)	18,289 (92.86%)	1,144 (95.02%)	0.004
Female	1,467 (7.02%)	1,407 (7.14%)	60 (4.98%)	
AJCC stage (N, %)				
Stage III	4,389 (21%)	4,165 (21.15%)	224 (18.6%)	0.036
Stage IVA-B	16,511 (79%)	15,531 (78.85%)	980 (81.4%)	
Primary tumor site (N, %)				
Oral cavity	13,142 (62.88%)	12,890 (65.44%)	252 (20.93%)	< 0.001
Oropharynx	2,794 (13.37%)	2,489 (12.64%)	305 (25.33%)	
Hypopharynx	3,853 (18.44%)	3,307 (16.79%)	546 (45.35%)	
Other	1,111 (5.32%)	1,010 (5.13%)	101 (8.39%)	
Histology				
Squamous cell carcinoma	20,251 (96.89%)	19,063 (96.79%)	1,188 (98.67%)	< 0.001
Others	649 (3.11%)	633 (3.21%)	16 (1.33%)	
CCI (Mean, SD)	1.05 ± 1.66	1.00 ± 1.61	1.83 ± 2.19	< 0.001
CCI category (N, %)				
0	11,573 (55.37%)	11,138 (56.55%)	435 (36.13%)	< 0.001
1	3,608 (17.26%)	3,370 (17.11%)	238 (19.77%)	
2+	5,719 (27.36%)	5,188 (26.34%)	531 (44.1%)	
Smoking (N, %)				
No	2,662 (12.74%)	2,469 (12.54%)	193 (16.03%)	< 0.001
Yes	13,505 (64.62%)	12,714 (64.55%)	791 (65.7%)	
Unknown	4,733 (22.65%)	4,513 (22.91%)	220 (18.27%)	
Betel nut chewing (N, %)				
No	5,277 (25.25%)	4,824 (24.49%)	453 (37.62%)	< 0.001
Yes	10,810 (51.72%)	10,289 (52.24%)	521 (43.27%)	
Unknown	4,813 (23.03%)	4,583 (23.27%)	230 (19.1%)	
Drinking (N, %)				
No	4,631 (22.16%)	4,337 (22.02%)	294 (24.42%)	0.001
Yes	11,451 (54.79%)	10,766 (54.66%)	685 (56.89%)	
Unknown	4,818 (23.05%)	4,593 (23.32%)	225 (18.69%)	
Diagnosis year (N, %)				
2009	2,243 (10.73%)	2,175 (11.04%)	68 (5.65%)	< 0.001
2010	2,399 (11.48%)	2,257 (11.46%)	142 (11.79%)	
2011	2,522 (12.07%)	2,361 (11.99%)	161 (13.37%)	
2012	2,480 (11.87%)	2,322 (11.79%)	158 (13.12%)	
2013	2,735 (13.09%)	2,574 (13.07%)	161 (13.37%)	
2014	2,823 (13.51%)	2,677 (13.59%)	146 (12.13%)	
2015	2,761 (13.21%)	2,587 (13.13%)	174 (14.45%)	
2016	2,937 (14.05%)	2,743 (13.93%)	194 (16.11%)	

Note: w/t, without; CCI = Charlson comorbidity index

Table 3 shows results from the multivariable logistic regression and Firth’s logistic regression for examining the factors associated with use of targeted therapy. Older patients with higher comorbidity were more likely to receive targeted therapy. The stage IVA-IVB group had 19% higher odds of receiving targeted therapy (OR [95% CI]: 1.19 [1.00-1.41]; *P* = 0.046). Patients with oropharynx and hypopharynx cancers were more likely to receive targeted therapy compared with those with oral cavity cancer (OR [95% CI] oropharynx, hypopharynx: 5.61 [4.64–6.76], 7.23 [6.12–8.55]; *P* < 0.001). Targeted therapy was significantly associated with radiotherapy but not with surgery or chemotherapy. History of alcohol drinking, betel nut chewing, and tobacco smoking seemed to have no significant association with targeted therapy. All results of Firth’s logistic regression were consistent to results of multivariable logistic regression.

**Table 3:** Results from multiple and Firth’s logistic regression examining factors associated with receiving targeted therapy treatment among locally advance head and neck cancer patients in 2009–2016

	Multiple logistic regression		Firth’s logistic regression	
	OR (95%CI)	*P*	OR (95%CI)	*P*
Age category				
≤ 44 (ref.)				
45–54	1.74 (1.26–2.40)	0.001	1.73 (1.25–2.38)	0.001
55–64	2.85 (2.08–3.91)	< 0.001	2.82 (2.06–3.86)	< 0.001
≥ 65	10.93 (7.99–14.96)	< 0.001	10.77 (7.89–14.71)	< 0.001
Sex				
Male (ref.)				
Female	0.58 (0.43–0.78)	< 0.001	0.58 (0.43–0.79)	< 0.001
Tumor stage				
III (ref.)				
IVA-IVB	1.19 (1.00-1.41)	0.046	1.19 (1.00-1.40)	0.047
Tumor site				
Oral cavity (ref.)				
Oropharynx	5.61 (4.64–6.76)	< 0.001	5.58 (4.63–6.72)	< 0.001
Hypopharynx	7.23 (6.12–8.55)	< 0.001	7.19 (6.08–8.49)	< 0.001
Others	2.76 (2.12–3.60)	< 0.001	2.76 (2.12–3.59)	< 0.001
CCI score category				
0 (ref.)				
1	1.20 (1.00-1.43)	0.047	1.20 (1.00-1.43)	0.046
≥ 2	1.37 (1.18–1.59)	< 0.001	1.37 (1.18–1.59)	< 0.001
Surgery				
No (ref.)				
Yes	0.51 (0.44–0.58)	< 0.001	0.51 (0.44–0.58)	< 0.001
Radiotherapy				
No (ref.)				
Yes	30.29 (14.32–64.05)	< 0.001	28.17 (13.71–57.87)	< 0.001
Chemotherapy				
No (ref.)				
Yes	0.51 (0.44–0.60)	< 0.001	0.51 (0.44–0.60)	< 0.001
Drinking				
No (ref.)				
Yes	1.13 (0.94–1.37)	0.185	1.13 (0.94–1.36)	0.187
Unknow	1.04 (0.44–2.46)	0.925	1.08 (0.47–2.52)	0.851
Betel nut chewing				
No (ref.)				
Yes	0.96 (0.81–1.14)	0.646	0.96 (0.81–1.14)	0.639
Unknow	1.07 (0.52–2.20)	0.859	1.09 (0.53–2.23)	0.815
Smoking				
No (ref.)				
Yes	1.00 (0.80–1.24)	0.982	1.00 (0.80–1.24)	0.977
Unknow	1.03 (0.31–3.35)	0.966	1.01 (0.31–3.24)	0.993
Diagnosis year				
2009 (ref.)				
2010	1.99 (1.45–2.74)	< 0.001	1.98 (1.45–2.72)	< 0.001
2011	2.73 (1.27–5.88)	0.010	2.83 (1.32–6.06)	0.007
2012	2.46 (1.13–5.32)	0.023	2.55 (1.19–5.48)	0.016
2013	1.98 (0.91–4.28)	0.083	2.06 (0.96–4.41)	0.064
2014	1.81 (0.83–3.91)	0.133	1.88 (0.87–4.03)	0.106
2015	2.16 (1.00-4.66)	0.050	2.24 (1.05–4.79)	0.037
2016	2.20 (1.02–4.74)	0.045	2.28 (1.06–4.88)	0.034

Note: OR = odds ratio; ref.=reference group; CCI = Charlson comorbidity index

We found one-year and long-term crude mortality rate ratio (MRR) for both all-cause mortality and cancer-specific mortality with respect to person-years and survival frequencies between targeted and nontargeted therapy groups. For example, the overall MRR of long-term all-cause mortality (MRR [95% CI]: 1.99 [1.86–2.12]) among those with targeted therapy was greater than those without targeted therapy (Supplementary eTables 12 and 13). We then further considered the effect of with or without receiving targeted therapies in addition to treatment modalities as a whole on patients’ all-cause, cancer-specific mortality among locally advanced head and neck cancer patients. As Table 4 shows, patients who received targeted therapy in addition to other treatment modalities had a greater risk of one-year and long-term all-cause mortality or cancer-specific mortality than those without receiving targeted therapy. Supplementary eTable 14 provides summarized results from cox proportional hazard regression models for the association between treatment modalities with targeted and nontargeted therapy and all-cause, cancer-specific mortality among locally advanced head and neck cancer patients (overall, by tumor stages, and cancer sites), and found consistent results.

**Table 4:** Results from Cox proportional hazard regression models for the association between treatment modalities with and without targeted therapy and all-cause, cancer-specific mortality among locally advanced head and neck cancer patients in 2009–2016

Variables	All-cause mortality				Cancer-specific mortality			
	One-year		Long-term		One-year		Long-term	
	HR (95% CI)	*P*	HR (95% CI)	*P*	HR (95% CI)	*P*	HR (95% CI)	*P*
Treatment modalities								
Surgery only (ref.)								
Surgery + RT	4.44 (3.29-6.00)	< 0.001	1.32 (1.18–1.47)	< 0.001	5.28 (3.75–7.43)	< 0.001	1.52 (1.34–1.73)	< 0.001
Surgery + RT + CT	9.38 (7.27–12.10)	< 0.001	1.95 (1.78–2.12)	< 0.001	11.32 (8.43–15.19)	< 0.001	2.37 (2.13–2.64)	< 0.001
Surgery + RT + CT + TT	12.33 (8.07–18.83)	< 0.001	2.48 (2.09–2.94)	< 0.001	14.20 (8.77–22.97)	< 0.001	3.14 (2.59–3.82)	< 0.001
RT only	6.05 (4.52–8.10)	< 0.001	2.50 (2.23–2.80)	< 0.001	6.64 (4.76–9.26)	< 0.001	2.74 (2.39–3.15)	< 0.001
RT + CT	9.98 (7.75–12.86)	< 0.001	2.54 (2.33–2.78)	< 0.001	12.42 (9.28–16.64)	< 0.001	3.19 (2.87–3.55)	< 0.001
RT + TT	10.84 (7.21–16.30)	< 0.001	3.20 (2.69–3.80)	< 0.001	11.80 (7.41–18.79)	< 0.001	3.77 (3.07–4.62)	< 0.001
RT + CT + TT	14.83 (10.66–20.63)	< 0.001	2.98 (2.62–3.38)	< 0.001	19.85 (13.72–28.71)	< 0.001	3.95 (3.40–4.58)	< 0.001
All other combinations	7.48 (5.74–9.75)	< 0.001	3.12 (2.82–3.45)	< 0.001	8.99 (6.63–12.19)	< 0.001	3.78 (3.35–4.26)	< 0.001
Age categories								
≤ 44 (ref.)								
45–54	1.08 (0.98–1.18)	0.105	0.99 (0.94–1.05)	0.854	1.06 (0.97–1.17)	0.207	0.97 (0.92–1.04)	0.399
55–64	1.00 (0.89–1.12)	0.946	1.00 (0.95–1.06)	0.981	0.98 (0.87–1.11)	0.783	0.94 (0.89–1.01)	0.080
≥ 65	1.32 (1.16–1.49)	< 0.001	1.47 (1.38–1.56)	< 0.001	1.23 (1.07–1.41)	0.003	1.29 (1.20–1.39)	< 0.001
Sex								
Male (ref.)								
Female	0.89 (0.79–1.01)	0.065	0.82 (0.75–0.88)	< 0.001	0.93 (0.82–1.06)	0.264	0.85 (0.77–0.93)	< 0.001
Tumor stage								
III (Ref)								
IVA-IVB	1.82 (1.61–2.06)	< 0.001	1.70 (1.61–1.80)	< 0.001	1.93 (1.68–2.22)	< 0.001	1.83 (1.72–1.94)	< 0.001
Tumor site								
Oral cavity (ref.)								
Oropharynx	0.59 (0.52–0.67)	< 0.001	0.83 (0.78–0.88)	< 0.001	0.53 (0.46–0.61)	< 0.001	0.70 (0.65–0.74)	< 0.001
Hypopharynx	0.80 (0.72–0.89)	< 0.001	1.11 (1.06–1.17)	< 0.001	0.75 (0.67–0.84)	< 0.001	1.03 (0.97–1.08)	0.307
Others	0.60 (0.52–0.70)	< 0.001	0.79 (0.72–0.86)	< 0.001	0.56 (0.48–0.66)	< 0.001	0.74 (0.66–0.82)	< 0.001
CCI categories								
0 (ref.)								
1	1.09 (1.01–1.18)	0.031	1.07 (1.01–1.12)	0.012	1.04 (0.96–1.13)	0.364	1.02 (0.96–1.08)	0.478
≥ 2	1.33 (1.22–1.46)	< 0.001	1.35 (1.30–1.41)	< 0.001	1.30 (1.18–1.44)	< 0.001	1.28 (1.22–1.34)	< 0.001
Alcohol history								
No (ref.)								
Yes	1.10 (1.01–1.19)	0.022	1.14 (1.08–1.20)	< 0.001	1.11 (1.02–1.22)	0.015	1.12 (1.05–1.19)	< 0.001
Unknow	1.29 (0.91–1.84)	0.147	1.21 (0.94–1.54)	0.135	1.03 (0.69–1.56)	0.872	1.08 (0.81–1.43)	0.610
Betel nut history								
No (ref.)								
Yes	0.91 (0.84–0.98)	0.017	0.96 (0.91–1.01)	0.122	0.92 (0.85-1.00)	0.055	0.99 (0.94–1.05)	0.788
Unknow	1.31 (0.94–1.82)	0.105	1.24 (0.98–1.57)	0.068	1.37 (0.96–1.95)	0.081	1.32 (1.02–1.72)	0.036
Cigarette history								
No (ref.)								
Yes	0.98 (0.88–1.08)	0.650	1.01 (0.94–1.08)	0.885	0.94 (0.85–1.05)	0.290	0.95 (0.88–1.02)	0.157
Unknow	0.86 (0.52–1.43)	0.558	1.07 (0.76–1.52)	0.694	1.05 (0.60–1.84)	0.871	1.12 (0.75–1.66)	0.585
Diagnosis year								
2009 (ref.)								
2010	1.07 (0.95–1.19)	0.259	1.05 (0.98–1.12)	0.203	1.08 (0.96–1.22)	0.219	1.06 (0.98–1.15)	0.162
2011	1.36 (1.00-1.85)	0.053	1.42 (1.14–1.77)	0.002	1.39 (1.00-1.93)	0.052	1.47 (1.15–1.88)	0.002
2012	1.32 (0.97–1.81)	0.077	1.36 (1.09–1.70)	0.007	1.44 (1.03–2.01)	0.031	1.41 (1.10–1.81)	0.007
2013	1.30 (0.96–1.78)	0.094	1.32 (1.06–1.65)	0.014	1.38 (0.99–1.92)	0.059	1.36 (1.06–1.75)	0.015
2014	1.20 (0.88–1.64)	0.249	1.32 (1.05–1.64)	0.015	1.28 (0.92–1.78)	0.146	1.36 (1.06–1.75)	0.015
2015	1.25 (0.92–1.70)	0.160	1.31 (1.05–1.64)	0.017	1.33 (0.95–1.84)	0.094	1.39 (1.09–1.79)	0.009
2016	1.21 (0.89–1.66)	0.225	1.27 (1.01–1.59)	0.038	1.28 (0.92–1.78)	0.148	1.35 (1.05–1.73)	0.019

Note: HR, hazard ratio; CI, confidence interval; ref., reference group; RT, radiotherapy; CT, chemotherapy; TT, targeted therapy; CCI, Charlson comorbidity index

## Discussion

This study utilized Taiwan’s National Health Insurance Research Database and Taiwan Cancer Registry to investigate treatment trends for locally advanced head and neck cancer (LAHNC) patients in Taiwan, both before and after the reimbursement of cetuximab in 2009. The study also examined the factors influencing treatment selection, as well as one-year and long-term mortality rates, among LAHNC patients who received targeted therapies versus those who did not. As financial reimbursement and clinical practices can vary across health systems, this research aimed to increase our understanding of treatment trends for new substitute targeted therapies and their impact on health outcomes in different healthcare settings.

We found an increasing trend of targeted therapy (cetuximab) after reimbursement in Taiwan, but the utilization rates were relatively low. Similar increasing trends were found in existing studies. For example, Schlichting et al. (2019) showed that cetuximab use in LAHNC patients significantly increased from 2004 to 2009 in the US, which approved cetuximab in 2006 (19% versus 1%; *P* for Cochran–Armitage trend tests ≤ 0.01) [14]. Yamada et al. (2019) found that cetuximab use dramatically increased in 2013 after the Japanese government approved cetuximab, but it remained stable, in which about 60% of patients received cisplatin and 15% cetuximab [16]. In contrast to the proportion of cetuximab use observed in other countries, our study revealed a remarkably low percentage (3.03%) of patients receiving cetuximab annually in Taiwan, which remained relatively stable from 2010 to 2016. This may be attributed to the significant reimbursement limitations in Taiwan and the higher medical expenses associated with cetuximab compared to the most commonly used systemic therapy regimen, cisplatin. Additionally, the limited evidence comparing cisplatin with cetuximab may have caused clinicians to be cautious when prescribing cetuximab in LAHNC patients in Taiwan[19].

In addition, the current study further summarized the most commonly used systemic agents were cisplatin plus 5-FU (plus docetaxel) in induction therapy and cisplatin in chemoradiotherapy, consistent with the NCCN guideline [4]. Lee et al.(2020) and Dansky et al.(2012) both showed similar treatment patterns. About half (48.7%) of the LAHNC patients received docetaxel plus cisplatin, followed by docetaxel plus cisplatin plus 5-FU (26.6%), and cisplatin plus 5-FU (17.1%). Cisplatin was still the most-used agent in the concurrent chemotherapy setting, with a proportion of 85.6% [26, 27].

With respect to the factors associated with receiving targeted therapy treatment among LAHNC patients, Yamada et al. (2019) that patients aged older than 66 years than younger than 65 years received cetuximab (18.6% versus 9.4%). The OR [95% CI] of 3.01[2.26–4.02] for cetuximab versus cisplatin comparing older to younger age was shown in multinomial logistic regression analysis[16]. Baxi et al.(2016) also found patients aged 70 to 79 years were more likely to receive concurrent chemoradiotherapy than patients aged younger than 70 years: OR [95% CI] 1.29 [1.02–1.64] [13]. The current study found older patients with more comorbid conditions and patients with hypopharynx and oropharynx cancers were more likely to receive targeted therapy. Specially, patients aged older than 65 years had 10.93 times the odds of receiving targeted therapy compared with those aged younger than 44 years. After its approval for reimbursement in Taiwan, cetuximab was primarily administered concurrently with radiotherapy in patients aged over 65 years (46.17%), who were more likely to be ineligible for cytotoxic chemotherapy. From the research results, it may be inferred that payment policies and clinical evidences may guide the utilization of drug prescriptions among LAHNC patients.

Existing literature found mixed results of overall survival outcomes between targeted therapy (cetuximab) and non-targeted therapy (cisplatin) [9, 17–20]. Our study found that patients who received targeted therapy controlling for other treatment modalities had a relative greater risk of mortality compared to those who did not receive targeted therapy. The results were consistent with Xiang et al. (2018), which used real world evidence from the Surveillance, Epidemiology, and End Results (SEERs)-Medicare database with large sample (n = 1,396) in the U.S., and a systematic review by Tang et al. (2020) from twenty-three studies with total of 8701 patients [17, 18]. One recent publication from Gebre-Medhin et al. (2021) reported results from a randomized controlled phase III study comparing treatment outcome and toxicity between RT with concomitant cisplatin versus cetuximab in HNSCC patients (n = 291) and found overall survival outcomes of cetuximab was not superior to cisplatin (adjusted hazard ratio1.63; 95% CI, 0.93 to 2.86) [9]. Administering cetuximab concurrently with radiation therapy may lead to improved survival outcomes by avoiding the harsh side effects associated with concurrent chemotherapy. This option could be considered for patients who are unable to tolerate cisplatin. However, additional research is necessary to identify specific subgroups of patients who could still benefit from concomitant cetuximab treatment.

To the best of our knowledge, ours was the a large population-based study from 2008 to 2016 investigating treatment trends of concomitant cetuximab and cisplatin, selection factors, and survival effects for patients with LAHNC in Taiwan. The study is valuable because it provides real world evidences of clinical practices from a whole population-based data from a detailed viewpoint. There were also some limitations in this study. First, due to the limitations of the NHIRD, which only includes reimbursement records, we were unable to gather adequate information on medications that were self-paid by the patients [21, 22]. As a result, we could not verify whether these treatments were utilized or if patients bore the costs themselves. Second, the cancer-specific mortality may be imprecise because the exact cause of death was not always recorded. Third, with the release of AJCC 8th edition in 2017, the Taiwan Cancer Registry began including human papillomavirus (HPV) status information. HPV has been linked to oropharyngeal cancer and is a reliable indicator of improved prognosis[5, 28]. Unfortunately, our study utilized data from 2008 to 2016, which predates the availability of HPV information. As a result, we were unable to determine the HPV status of the patients included in our analysis. Finally, while the findings of our study may not be generalizable to other countries, they do offer valuable real-world evidence from an Asian population within the Taiwanese healthcare system. As such, they warrant attention and can contribute to a more comprehensive understanding of treatment outcomes in LAHNC patient population.

## Conclusions

Our study has revealed an increasing trend in the utilization of cetuximab as targeted therapy in Taiwan after its reimbursement; however, the overall usage rates were still relatively low. With a large population-based patient cohort, we have observed that patients who received concomitant targeted therapy with cetuximab, while controlling for other treatment modalities, had a higher risk of mortality compared to those who received cisplatin therapy. These findings suggest that cisplatin may be the preferred chemotherapeutic agent. Further research is necessary to identify specific patient subgroups that could still benefit from concomitant cetuximab treatment.

## Electronic supplementary material

Below is the link to the electronic supplementary material.

Supplementary Material 1

## Data Availability

The data that support the findings of this study are available from Taiwan’s Health and Welfare Data Science Center, Ministry of Health and Welfare, but restrictions apply to the availability of these data, which were used under license for the current study, and so are not publicly available. Data are however available from the corresponding author upon reasonable request and with permission of Taiwan’s Health and Welfare Data Science Center, Ministry of Health and Welfare.
