# Supplementary material for: Treatment patterns of targeted and nontargeted therapies and survival effects in patients with locally advanced head and neck cancer in Taiwan
Source: BMC Cancer. 2023 Jun 20;23:567. doi: 10.1186/s12885-023-11061-4 (PMC10283176; doi:10.1186/s12885-023-11061-4)
Supplement: Supplementary file 1 — Supplementary Material 1 [file 12885_2023_11061_MOESM1_ESM.docx]

**Supplementary Digital Content**

This appendix is provided by the authors to give readers additional information about this manuscript. Supplement to: “Treatment Patterns of Targeted and Nontargeted Therapies and Survival Effects in Patients with Locally Advanced Head and Neck Cancer in Taiwan”

**Supplementary eTable 1.** Coding algorithms for locally advanced head and neck cancer

| **ICD-O-FT** | **ICD-O-3** | **Topography** | **Classification** |
| --- | --- | --- | --- |
| 1400 | C000 | External upper lip | Oral cavity |
| 1401 | C001 | External lower lip |  |
| 1403 | C002 | External lip, NOS |  |
| 1404 | C003 | Mucosa of upper lip |  |
| 1405 | C004 | Mucosa of lower lip |  |
| 1406 | C005 | Mucosa of lip, NOS |  |
| 1408 | C006 | Commissure of lip |  |
| 1409 | C008 | Overlapping lesion of lip |  |
| 1409 | C009 | Lip, NOS |  |
| 1410 | C019 | Base of tongue, NOS |  |
| 1411 | C020 | Dorsal surface of tongue, NOS |  |
| 1412 | C021 | Border of tongue |  |
| 1413 | C022 | Ventral surface of tongue, NOS |  |
| 1414 | C023 | Anterior 2/3 of tongue, NOS |  |
| 1416 | C024 | Lingual tonsil |  |
| 1415 | C028 | Junctional zone of tongue |  |
| 1418 | C028 | Overlapping lesion of tongue |  |
| 1419 | C029 | Tongue, NOS |  |
| 1430 | C030 | Upper Gum |  |
| 1431 | C031 | Lower gum |  |
| 1438 | C038 | Overlapping lesion of gum |  |
| 1439 | C039 | Gum, NOS |  |
| 1440 | C040 | Anterior floor of mouth |  |
| 1441 | C041 | Lateral floor of mouth |  |
| 1448 | C048 | Overlapping lesion of floor of mouth |  |
| 1449 | C049 | Floor of mouth, NOS |  |
| 1452 | C050 | Hard palate |  |
| 1453 | C051 | Soft palate, NOS |  |
| 1454 | C052 | Uvula |  |
| 1455 | C058 | Overlapping lesion of palate |  |
| 1455 | C059 | Palate, NOS |  |
| 1450 | C060 | Cheeck mucosa |  |
| 1451 | C061 | Vestibule of mouth |  |
| 1456 | C062 | Retromolar area |  |
| 1458 | C068 | Overlapping lesion of other and unspecified parts of mouth |  |
| 1459 | C069 | Mouth, NOS |  |
| 1420 | C079 | Parotid gland | Other |
| 1421 | C080 | Submandibular gland |  |
| 1422 | C081 | Sublingual gland |  |
| 1428 | C088 | Overlapping lesion of major salivary glands |  |
| 1429 | C089 | Major salivary gland, NOS |  |
| 1461 | C090 | Tonsillar fossa | Oropharynx |
| 1462 | C091 | Tonsillar pillar |  |
| 1460 | C098 | Overlapping lesion of tonsil |  |
| 1460 | C099 | Tonsil, NOS |  |
| 1463 | C100 | Vallecula |  |
| 1464 | C101 | Anterior surface of epiglottis |  |
| 1466 | C102 | Lateral wall of oropharynx |  |
| 1467 | C103 | Posterior wall of oropharynx |  |
| 1468 | C104 | Branchial cleft |  |
| 1465 | C108 | Overlapping lesions of oropharynx |  |
| 1469 | C109 | Oropharynx, NOS |  |
| 1481 | C129 | Pyriform sinus | Hypopharynx |
| 1480 | C130 | Postcricoid region |  |
| 1482 | C131 | Hypopharyngeal aspect of aryepiglottic fold |  |
| 1483 | C132 | Posterior wall of hypopharynx |  |
| 1488 | C138 | Overlapping lesion of hypopharynx |  |
| 1489 | C139 | Hypopharynx, NOS |  |
| 1490 | C140 | Pharynx, NOS | Other |
| 1491 | C142 | Waldeyer ring |  |
| 1498 | C148 | Overlapping lesion of lip, oral cavity and pharynx |  |
| 1610 | C320 | Glottis | Other (larynx) |
| 1611 | C321 | Supraglottis |  |
| 1612 | C322 | Subglottis |  |
| 1613 | C323 | Laryngeal cartilage |  |
| 1618 | C328 | Overlapping lesion of larynx |  |
| 1619 | C329 | Larynx, NOS |  |

**Supplementary eTable 2.** Coding algorithms for squamous cell carcinoma

| **M-code** | **ICD-O-3 Morphology** |
| --- | --- |
| 80323 | Spindle cell carcinoma, NOS |
| 80333 | Pseudosarcomatous carcinoma |
| 80333 | Sarcomatoid carcinoma |
| 80502 | Papillary carcinoma in situ |
| 80503 | Papillary carcinoma, NOS |
| 80513 | Verrucous carcinoma, NOS |
| 80513 | Verrucous epidermoid carcinoma |
| 80513 | Verrucous squamous cell carcinoma |
| 80522 | Papillary squamous cell carcinoma, non-invasive |
| 80522 | Papillary squamous cell carcinoma in situ |
| 80523 | Papillary squamous cell carcinoma |
| 80523 | Papillary epidermoid carcinoma |
| 80543 | Warty carcinoma |
| 80543 | Condylomatous carcinoma |
| 80543 | Warty-basaloid carcinoma |
| 80702 | Squamous cell carcinoma in situ, NOS |
| 80702 | Epidermoid carcinoma in situ, NOS |
| 80702 | Intraepidermal carcinoma, NOS |
| 80702 | Intraepithelial squamous cell carcinoma |
| 80703 | Squamous cell carcinoma, NOS |
| 80703 | Squamous cell carcinoma, usual type |
| 80703 | Epidermoid carcinoma, NOS |
| 80703 | Squamous carcinoma |
| 80703 | Squamous cell epithelioma |
| 80712 | Differentiated intraepithelial neoplasia |
| 80712 | Differentiated penile intraepithelial neoplasia (PeIN) |
| 80712 | Differentiated vulva intraepithelial neoplasia (VIN) |
| 80713 | Squamous cell carcinoma, keratinizing, NOS |
| 80713 | Epidermoid carcinoma, keratinizing |
| 80713 | Squamous cell carcinoma, large cell, keratinizing |
| 80713 | Keratoacanthoma |
| 80723 | Squamous cell carcinoma, large cell, nonkeratinizing, NOS |
| 80723 | Epidermoid carcinoma, large cell, nonkeratinizing |
| 80723 | Squamous cell carcinoma, nonkeratinizing, NOS |
| 80733 | Squamous cell carcinoma, small cell, nonkeratinizing |
| 80733 | Epidermoid carcinoma, small cell, nonkeratinizing |
| 80743 | Squamous cell carcinoma, spindle cell |
| 80743 | Epidermoid carcinoma, spindle cell |
| 80743 | Squamous cell carcinoma, sarcomatoid |
| 80743 | Pseudovascular squamous cell carcinoma |
| 80753 | Squamous cell carcinoma, adenoid |
| 80753 | Squamous cell carcinoma, acantholytic |
| 80753 | Squamous cell carcinoma, pseudoglandular |
| 80762 | Squamous cell carcinoma in situ with questionable stromal invasion |
| 80762 | Epidermoid carcinoma in situ with questionable stromal invasion |
| 80763 | Squamous cell carcinoma, microinvasive |
| 80772 | Squamous intraepithelial neoplasia, high grade |
| 80772 | Squamous intraepithelial neoplasia, grade III |
| 80772 | Squamous dysplasia, high grade |
| 80772 | High grade intra-epithelial lesion |
| 80772 | Anal intraepithelial neoplasia, grade III |
| 80772 | AIN III |
| 80772 | Cervical intraepithelial neoplasia, grade III |
| 80772 | CIN III, NOS |
| 80772 | CIN III with severe dysplasia |
| 80772 | Esophageal squamous intraepithelial neoplasia (dysplasia), high grade |
| 80772 | Vaginal intraepithelial neoplasia, grade III |
| 80772 | VAIN III |
| 80772 | Vulvar intraepithelial neoplasia, grade III |
| 80772 | VIN III |
| 80783 | Squamous cell carcinoma with horn formation |
| 80802 | Queyrat erythroplasia |
| 80812 | Bowen disease |
| 80812 | Intraepidermal squamous cell carcinoma, Bowen type |
| 80823 | Lymphoepithelial carcinoma |
| 80823 | Lymphoepithelioma |
| 80823 | Lymphoepithelioma-like carcinoma |
| 80823 | Schmincke tumor |
| 80833 | Papillary-basaloid carcinoma |
| 80833 | Basaloid squamous cell carcinoma |
| 80843 | Squamous cell carcinoma, clear cell type |
| 80853 | Squamous cell carcinoma, HPV-positive |
| 80863 | Squamous cell carcinoma, HPV-negative |
| 80903 | Basal cell carcinoma, NOS |
| 80903 | Basal cell epithelioma |
| 80903 | Rodent ulcer |
| 80903 | Pigmented basal cell carcinoma |
| 80903 | Basal cell carcinoma with adnexal differentiation |
| 85603 | Adenosquamous carcinoma |
| 85603 | Mixed adenocarcinoma and epidermoid carcinoma |
| 85603 | Mixed adenocarcinoma and squamous cell carcinoma |
| 85603 | Squamoid eccrine ductal carcinoma |

**Supplementary eTable 3.** Coding algorithms and weight for the Charlson comorbidity index

| **Comorbid conditions** | **Weight** | **Codes** | |
| --- | --- | --- | --- |
|  |  | **ICD-9-CM** | **ICD-10-CM** |
| Myocardial infarction | 1 | 410.x, 412.x | I21.x, I22.x, I25.2 |
| Congestive heart failure | 1 | 398.91,402.01,402.11,402.91,404.01,404.03,404.11, 404.13,404.91, 404.93,425.4-425.9, 428.x | I09.9, I11.0, I13.0, I13.2, I25.5, I42.0, I42.5–I42.9, I43.x, I50.x, P29.0 |
| Peripheral vascular disease | 1 | 093.0, 437.3, 440.x, 441.x,443.1-443.9, 47.1, 557.1,557.9, V43.4 | I70.x, I71.x, I73.1, I73.8, I73.9, I77.1, I79.0, I79.2, K55.1, K55.8, K55.9, Z95.8, Z95.9 |
| Cerebrovascular disease | 1 | 362.34, 430.x-438.x | G45.x, G46.x, H34.0, I60.x–I69.x |
| Dementia | 1 | 290.x, 294.1, 331.2 | F00.x–F03.x, F05.1, G30.x, G31.1 |
| Chronic pulmonary disease | 1 | 416.8, 416.9, 490.x–505.x,506.4, 508.1, 508.8 | I27.8, I27.9, J40.x–J47.x, J60.x–J67.x, J68.4, J70.1, J70.3 |
| Rheumatic disease | 1 | 446.5, 710.0–710.4, 714.0–714.2, 714.8, 725.x | M05.x, M06.x, M31.5, M32.x–M34.x, M35.1, M35.3, M36.0 |
| Peptic ulcer disease | 1 | 531.x–534.x | K25.x–K28.x |
| Mild liver disease | 1 | 070.22,070.23,070.32, 070.33,070.44,070.54, 070.6, 070.9, 570.x, 571.x, 573.3,573.4, 573.8, 573.9, V42.7 | B18.x, K70.0–K70.3, K70.9, K71.3–K71.5, K71.7, K73.x, K74.x, K76.0, K76.2–K76.4, K76.8, K76.9, Z94.4 |
| Diabetes without chronic  complication | 1 | 250.0–250.3,250.8, 250.9 | E10.0, E10.1, E10.6, E10.8, E10.9, E11.0, E11.1, E11.6, E11.8, E11.9, E12.0, E12.1, E12.6, E12.8, E12.9, E13.0, E13.1, E13.6, E13.8, E13.9, E14.0, E14.1, E14.6, E14.8, E14.9 |
| Diabetes with chronic complication | 2 | 250.4–250.7 | E10.2–E10.5, E10.7, E11.2-E11.5, E11.7, E12.2–E12.5, E12.7, E13.2–E13.5, E13.7, E14.2-E14.5, E14.7 |
| Hemiplegia or paraplegia | 2 | 334.1, 342.x, 343.x, 344.0-344.6, 344.9 | G04.1, G11.4, G80.1, G80.2, G81.x, G82.x, G83.0-G83.4, G83.9 |
| Renal disease | 2 | 403.01, 403.11, 403.91, 404.02, 404.03, 404.12, 404.13,404.92, 404.93, 582.x, 583.0-583.7, 585.x, 586.x,588.0, V42.0, V45.1, V56.x | I12.0, I13.1, N03.2–N03.7, N05.2–N05.7, N18.x, N19.x, N25.0, Z49.0–Z49.2, Z94.0, Z99.2 |
| Any malignancy, including lymphoma and leukemia, except malignant neoplasm of skin | 2 | 140.x–172.x, 174.x–195.8, 200.x–208.x, 238.6 | C00.x–C26.x, C30.x–C34.x, C37.x–C41.x, C43.x, C45.x–C58.x, C60.x–C76.x, C81.x–C85.x, C88.x, C90.x–C97.x |
| Moderate or severe liver disease | 3 | 456.0–456.2, 572.2–572.8 | I85.0, I85.9, I86.4, I98.2, K70.4, K71.1, K72.1, K72.9, K76.5, K76.6, K76.7 |
| Metastatic solid tumor | 6 | 196.x–199.x | C77.x–C80.x |
| AIDS/HIV | 6 | 042.x–044.x | B20.x–B22.x, B24.x |

AIDS/HIV: acquired immune deficiency syndrome/human immunodeficiency virus.

**Supplementary eTable 4.** NHI code and procedure name for surgery and radiotherapy

| **NHI billing code** | **Procedure name** |
| --- | --- |
| **Radiotherapy** | |
| 36011B | Linear accelerator teletherapy, each simple portal |
| 36012B | Linear accelerator teletherapy, each complex portal |
| 36013B | Emergent linear accelerator teletherapy, each portal |
| **Surgery** | |
| 62001C | Excision of facial skin and subcutaneous tumor - within 1cm in diameter |
| 62002C | Excision of facial skin and subcutaneous tumor - 1cm to 2cm in diameter |
| 62003C | Excision of facial skin tumor - over 2cm in diameter |
| 62010C | Excision of skin or subcutaneous tumor (Except face) － within 2cm |
| 62011C | Excision of skin or subcutaneous tumor (Except face) － 2 to 4 cm |
| 62012C | Excision of skin or subcutaneous tumor (Except face) - 4 to 10cm |
| 64100B | Extirpation of benign tumors on palate or tongue |
| 64116C | Benign neck mass excision (simple) |
| 64149B | Excision operation of malignant tumor of upper jaw with lymphadenectomy |
| 64150B | Excision operation of malignant tumor of upper jaw with neck dissection |
| 64151B | Excision operation of malignant tumor of lower jaw with lymphadenectomy |
| 64152B | Excision operation of malignant tumor of lower jaw with neck dissection |
| 65036B | Excision of nasal tumor with skin graft |
| 65041B | Excision of intranasal tumor |
| 65044B | Excision of tumor from frontal sinus |
| 65045B | Excision of tumor from maxillary sinus |
| 65046B | Excision of tumor from ethmoidal sinus |
| 65050B | Excision of nasopharyngeal tumor |
| 65053B | Excision of nasopalatine cyst |
| 65071B | Lateral rhinotomy with facial bone reposition |
| 66002B | Simple laryngoscopy |
| 66012B | Laryngectomy without neck dissection |
| 66013B | Laryngectomy with radical neck dissection |
| 66016B | Vertical (lateral/anterior) partial laryngectomy |
| 66017B | Radical neck dissection |
| 66023B | Laryngopharyngectomy |
| 66031C | Laryngo micro-surgery with CO2 laser |
| 71001B | Oral tumor or oropharynx excision |
| 71004B | Partial/wedge glossectomy |
| 71007C | Lingual tonsillectomy |
| 71009C | Cryotherapy for tonsillar |
| 71012B | Oral tumor or oropharynx excision with radical neck dissection |
| 71013B | Tongue cancer excision with lymphadenectomy & radical neck dissection |
| 71015B | Excision of parotid tumor |

**Supplementary eTable 5**. Code for antineoplastic agents

| **Drug** | **ATC code** | **NHI code** |
| --- | --- | --- |
| Cisplatin | L01XA01 | B012020248, B012184229, B016417229, B017805238, B017805248, B017805255, B018869238, B018873238, B018873255, B019333229, B019333248, B020193229, B020193263, B020221248, B020937238, B020937255, B020937263, B021782229, B021782248, B021782255, B022602238, B022602255, B024970238, B024970255, B025744229, B025744248, BC18873238, BC18873255, BC21782248, BC21782255, BC24970238, BC24970255, BC25744229, BC25744248, BC27762229, BC27762248 |
| Carboplatin | L01XA02 | BC21701235, BC217012AP, BC24074235, BC240742AP, BC24804235, BC248042AP, BC25626235, BC256262AP, BC26387235, BC27083235, BC270832AP |
| Cetuximab | L01XC06 | K000819248, K000877238, KC00877238 |
| Fluorouracil (5-FU) | L01BC02 | A009964221, AA58033238, AA58033248, AA58033255, AC58033238, AC58033248, AC58033255, AC58842238, B011924200, B013291221, B013603221, B019186221, B019186229, B020406229, B020406238, B020406255, B020793229, B020807221, B020807229, B020807238, B021121221, B021689221, B021689229, B021689248, B021720229, B021720238, B021720255, B023404221, B023404229,B023404238, BC21689221, BC21689229, BC21689248, BC23404221, BC23404229, BC23404238, BC26372221, BC26372229, BC26676229, BC26676238, BC26676255,BC27435221, BC27435229, BC27435238, BC27435248, BC27435255, X000124255, B017460338, B023514338 |
| Uracil-Tegafur (URACIL-TEGAFUR) | L01BC53 | A043698100, A049563100, AC43698100, AC49563100, B023484100, BC23484100 |
| Paclitaxel | L01CD01 | A044466221, A044466229, A044466235, A044466240, A044466248, A0444662E2, A0444662FG, A044601221, A044601229, A0446012E2, A0446012FG, A044611221, A045331221, A045331229, A045331235, A045331240, A045331248, A0453312E2, A046429221, AB44466221, AB44466229, AB44466235, AB44466240, AB44466248, AB444662E2, AB444662FG, AC44601221, AC44601229, AC44601240, AC446012FG, AC45331221, AC45331229, AC45331235, AC45331240, AC45331248, AC453312E2, B021157221, B0211572E2, B022400221, B022400240, B023396221, B023396240, B024463221, B024463240, B024463248, B0244632E2, B024476221, B024476240, B024476248, B0244762E2, B024738221, B024738248, B0247382BE, B0247382FG, BB21157221, BB211572E2, BC21157221, BC211572E2, BC23396221, BC23396240, BC23396248, BC233962E2, BC24738221, BC24738248, BC247382BE, BC247382FG, BC27434221, BC27434240, BC27434248, BC27434255, BC27434296, BC274342E2, BC27504221, BC27504248, BC275042E2 |
| Docetaxel | L01CD02 | A047882206, A047882212, AA55113206, AA55113212, AA56672206, AA56672212, AB47882206, AB47882212, AB55113206, AB55113212, AB56672206, AB56672212, AB57240209, AB57240219, AB58065209, AC57240209, AC57240219, AC57971209, AC57971219, AC58065209, AC58066209, AC58066219, AC58581209, AC58581219, AC58581223, B022048206, B022048212, B025055206, B025055212, B025289209, B025289219, B025558212, B025558226, BB25600212, BB25600226, BC25289209, BC25289219, BC25558212, BC25558226, BC26334209, BC26334219, BC27138206, BC27138212, BC27525209, BC27525219, BC27712209, BC27712219, BC27786209, BC27786219 |
| Cyclophosphamide | L01AA01 | B006463255, B006465277, B006915100, B012601100, B013563277, B013680263, B013687255, B016501263, B016517255, B016754100, B016870277, B017105209, B021304277, B021304209, B021304263, BC12601100, BC16754100, BC21304263, BC21304277 |
| Ifosfamide | L01AA06 | B018479212, B018480209, B018481277, BC18479212 |
| Methotrexate | L01BA01 | A008260212, A009101100, A025520100, AC59414212, AC59414226, AC59414238, AC59414245, B008678214, B008859212, B008859238, B008859240, B008859245, B008859263, B009144209, B011734212, B011734248, B011770212, B013232229, B013232299, B015981212, B015981226, B015983212, B016194100, B016195100, B016410248, B016419240, B017549212, B017549245, B017551245, B018876221, B018876229, B018876248, B018878221, B019707229, B019707299, B019712212, B019712238, B020440100, B020564212, B020643229, B020643248, B020808212, B020808238, B021051248, B021688212, B021696212, B021713229, B021722212, B021722238, B022132229, B022726100, BC16194100, BC18876221, BC18876229, BC18876248, BC18878221, BC21696212, BC21713229, BC22132229, BC22726100, X000115221, X000119238 |
| Vincristine | L01CA02 | A029529209, B009001209, B009289209, B014434209, B014436209, B014436229, B014444229, B015804209, B016222209, B016222212, B016422209, B019708209, B019708212, B020285209, B021719209, B021719212, B022047209, B022047212, B022167209, B022167212, B022167221, BC22047209, X000098209 |
| Doxorubicin | L01DB01 | A036418229, AA36418229, B012027229, B018174229, B018174248, B018580229, B018580299, B019117221, B019117248, B019443248, B019444229, B020335221, B020335229, B020335240, B021576221, B021576240, B021576248, B021718221, B021718229, B021718240, B022174248, B022175229, B025840221, B025840240, BB26026221, BB26026229, BB26026240, BB26026248, BC18580229, BC21576221, BC21576240, BC22712221, BC25808221, BC25808229, BC25808240, BC25840221, BC26026221, BC26026229, BC26026240, BC26026248, BC26572221, BC26572240 |
| Epirubicin | L01DB03 | A047003221, A047003229, A047003240, A047552229, A047552240, A047557229, A048329248, AB47003221, AB47003229, AB47003240, AB47557229, AB48329248, AC47003221, AC47003240, AC57985221, AC57985229, AC57985240, B014605248, B014606229, B019119248, B019120229, B019120248, B019120299, B020982229, B020982240, B024427221, B024427240, B024762221, B024762240, B025504221, B025504240, B025515221, B025515240, BB25968221, BB25968240, BC19119248, BC19120229, BC22695221, BC22695240, BC24427221, BC24427240, BC24762221, BC24762240, BC25515221, BC25515240, BC25968221, BC25968240, BC26699221, BC26699240 |
| Bleomycin | L01DC01 | B019124235, B019191221, B021837235, B023780235, B025353235, BC19124235, BC19191221 |
| Hydroxyurea | L01XX05 | B017468100, B020757100, B023135100, BC23135100, X000211100 |

**Supplementary eTable 6**. Subgroup analysis by sex in locally advanced head and neck cancer patients

|  | **Male** | | | **Female** | | | **Between-group difference *P*** |
| --- | --- | --- | --- | --- | --- | --- | --- |
|  | **Nontargeted therapy group** | **Targeted  therapy group** | ***P*** | **Nontargeted therapy group** | **Targeted therapy group** | ***P*** |  |
| **N** | **18,289** | **1,144** |  | **1,407** | **60** |  |  |
| Age category (N, %) |  |  |  |  |  |  |  |
| ≤ 44 years | 3,132 (17.13%) | 42 (3.67%) | **<0.001** | 173 (12.3%) | 5 (8.33%) | **<0.001** | **0.101** |
| 45-54 years | 6,672 (36.48%) | 217 (18.97%) |  | 380 (27.01%) | 5 (8.33%) |  |  |
| 55-64 years | 5,654 (30.91%) | 294 (25.7%) |  | 393 (27.93%) | 16 (26.67%) |  |  |
| ≥ 65 years | 2,831 (15.48%) | 591 (51.66%) |  | 461 (32.76%) | 34 (56.67%) |  |  |
| AJCC stage (N, %) |  |  |  |  |  |  |  |
| Stage III | 3,771 (20.62%) | 207 (18.09%) | **0.040** | 394 (28%) | 17 (28.33%) | **0.955** | **0.349** |
| Stage IVA-B | 14,518 (79.38%) | 937 (81.91%) |  | 1,013 (72%) | 43 (71.67%) |  |  |
| Primary tumor site (N, %) |  |  |  |  |  |  |  |
| Oral cavity | 11,976 (65.48%) | 233 (20.37%) | **<0.001** | 914 (64.96%) | 19 (31.67%) | **<0.001** | **0.783** |
| Oropharynx | 2,260 (12.36%) | 280 (24.48%) |  | 229 (16.28%) | 25 (41.67%) |  |  |
| Hypopharynx | 3,235 (17.69%) | 534 (46.68%) |  | 72 (5.12%) | 12 (20%) |  |  |
| Other | 818 (4.47%) | 97 (8.48%) |  | 192 (13.65%) | 4 (6.67%) |  |  |
| CCI category (N, %) |  |  |  |  |  |  |  |
| 0 | 10,436 (57.06%) | 416 (36.36%) | **<0.001** | 702 (49.89%) | 19 (31.67%) | **0.021** | **0.101** |
| 1 | 3,085 (16.87%) | 222 (19.41%) |  | 285 (20.26%) | 16 (26.67%) |  |  |
| 2+ | 4,768 (26.07%) | 506 (44.23%) |  | 420 (29.85%) | 25 (41.67%) |  |  |
| Surgery | 10,708 (58.55%) | 386 (33.74%) | **<0.001** | 721 (51.24%) | 13 (21.67%) | **<0.001** | **0.013** |
| Radiotherapy | 15,115 (82.65%) | 1,137 (99.39%) | **<0.001** | 1,156 (82.16%) | 60 (100%) | **<0.001** | **0.006** |
| Chemotherapy | 13,830 (75.62%) | 802 (70.1%) | **<0.001** | 937 (66.6%) | 35 (58.33%) | **0.185** | **0.012** |

Note:AJCC, American Joint Committee on Cancer; CCI = Charlson comorbidity index.

**Supplementary eTable 7.** Treatment modalities between LAHNC patients who did and did not receive targeted therapy (by tumor sites and cancer stages)

| Treatment modalities | Overall | | | | Stage III | | | | Stage IVA-IVB | | | |
| --- | --- | --- | --- | --- | --- | --- | --- | --- | --- | --- | --- | --- |
|  | All | Nontargeted therapy | Targeted  therapy | *P* | All | Nontargeted therapy | Targeted  therapy | *P* | All | Nontargeted therapy | Targeted  therapy | *P* |
| **Overall** | **20,900** | **19,696** | **1,204** |  | **4,389** | **4,165** | **224** |  | **16,511** | **15,531** | **980** |  |
| Any surgery | 11,828 (56.59%) | 11,429  (58.03%) | 399 (33.14%) | <0.001 | 2,829 (64.46%) | 2,755  (66.15%) | 74 (33.04%) | <0.001 | 8,999 (54.5%) | 8,674  (55.85%) | 325 (33.16%) | <0.001 |
| Any radiotherapy | 17,468 (83.58%) | 16,271  (82.61%) | 1,197 (99.42%) | <0.001 | 3,212 (73.18%) | 2,988  (71.74%) | 224  (100%) | <0.001 | 14,256 (86.34%) | 13,283  (85.53%) | 973 (99.29%) | <0.001 |
| Any chemotherapy | 15,604 (74.66%) | 14,767  (74.97%) | 837 (69.52%) | <0.001 | 2,500 (56.96%) | 2,378  (57.09%) | 122 (54.46%) | 0.439 | 13,104 (79.37%) | 12,389  (79.77%) | 715 (72.96%) | <0.001 |
| **Oral Cavity** | **13,142** | **12,890** | **252** |  | **2,909** | **2,867** | **42** |  | **10,233** | **10,023** | **210** |  |
| Any surgery | 8,464 (64.4%) | 8,392  (65.1%) | 72  (28.57%) | <0.001 | 2,183 (75.04%) | 2,169  (75.65%) | 14 (33.33%) | <0.001 | 6,281 (61.38%) | 6,223  (62.09%) | 58 (27.62%) | <0.001 |
| Any radiotherapy | 10,287 (78.28%) | 10,039 (77.88%) | 248 (98.41%) | <0.001 | 1,873 (64.39%) | 1,831  (63.86%) | 42  (100%) | <0.001 | 8,414 (82.22%) | 8,208  (81.89%) | 206 (98.1%) | <0.001 |
| Any chemotherapy | 9,098 (69.23%) | 8,914  (69.15%) | 184 (73.02%) | 0.188 | 1,456 (50.05%) | 1,429  (49.84%) | 27 (64.29%) | 0.063 | 7,642 (74.68%) | 7,485  (74.68%) | 157 (74.76%) | 0.978 |
| **Oropharynx** | **2,794** | **2,489** | **305** |  | **435** | **390** | **45** |  | **2,359** | **2,099** | **260** |  |
| Any surgery | 907  (32.46%) | 842  (33.83%) | 65  (21.31%) | <0.001 | 140 (32.18%) | 130  (33.33%) | 10 (22.22%) | 0.131 | 767  (32.51%) | 712  (33.92%) | 55 (21.15%) | <0.001 |
| Any radiotherapy | 2,633 (94.24%) | 2,328  (93.53%) | 305  (100%) | <0.001 | 399 (91.72%) | 354  (90.77%) | 45 (100%) | 0.039 | 2,234 (94.7%) | 1,974  (94.04%) | 260  (100%) | <0.001 |
| Any chemotherapy | 2,500 (89.48%) | 2,286  (91.84%) | 214 (70.16%) | <0.001 | 357 (82.07%) | 338  (86.67%) | 19 (42.22%) | <0.001 | 2,143 (90.84%) | 1,948  (92.81%) | 195  (75%) | <0.001 |
| **Hypopharynx** | **3,853** | **3,307** | **546** |  | **621** | **530** | **91** |  | **3,232** | **2,777** | **455** |  |
| Any surgery | 1,971 (51.15%) | 1,750  (52.92%) | 221 (40.48%) | <0.001 | 349  (56.2%) | 313  (59.06%) | 36 (39.56%) | 0.001 | 1,622 (50.19%) | 1,437  (51.75%) | 185 (40.66%) | <0.001 |
| Any radiotherapy | 3,556 (92.29%) | 3,013  (91.11%) | 543 (99.45%) | <0.001 | 567  (91.3%) | 476  (89.81%) | 91 (100%) | 0.001 | 2,989 (92.48%) | 2,537  (91.36%) | 452 (99.34%) | <0.001 |
| Any chemotherapy | 3,293 (85.47%) | 2,908  (87.93%) | 385 (70.51%) | <0.001 | 484 (77.94%) | 429  (80.94%) | 55 (60.44%) | <0.001 | 2,809 (86.91%) | 2,479  (89.27%) | 330 (72.53%) | <0.001 |
| **Other** | **1,111** | **1,010** | **101** |  | **424** | **378** | **46** |  | **687** | **632** | **55** |  |
| Any surgery | 486  (43.74%) | 445  (44.06%) | 41 (40.59%) | 0.503 | 157 (37.03%) | 143  (37.83%) | 14 (30.43%) | 0.327 | 329  (47.89%) | 302  (47.78%) | 27 (49.09%) | 0.852 |
| Any radiotherapy | 992  (89.29%) | 891  (88.22%) | 101 (100%) | <0.001 | 373 (87.97%) | 327  (86.51%) | 46  (100%) | 0.008 | 619  (90.1%) | 564  (89.24%) | 55  (100%) | 0.010 |
| Any chemotherapy | 713  (64.18%) | 659  (65.25%) | 54 (53.47%) | 0.019 | 203 (47.88%) | 182  (48.15%) | 21 (45.65%) | 0.749 | 510  (74.24%) | 477  (75.47%) | 33  (60%) | 0.012 |

**Supplementary eTable 8**. Treatment modalities by the tumor site and cancer stage in the nontargeted therapy group

| **Treatment modalities, N (%)** | **All** | **Stage III** | **Stage IVA-IVB** |
| --- | --- | --- | --- |
| **Overall** | **19,696** | **4,165** | **15,531** |
| Surgery only | 1,980 (10.05%) | 932 (22.38%) | 1,048 (6.75%) |
| Chemotherapy only | 1,028 (5.22%) | 346 (8.31%) | 682 (4.39%) |
| Surgery + radiotherapy | 1,921 (9.75%) | 509 (12.22%) | 1,412 (9.09%) |
| Radiotherapy + chemotherapy | 6,259 (31.78%) | 942 (22.62%) | 5,317 (34.23%) |
| Surgery + chemotherapy + radiotherapy | 7,063 (35.86%) | 1,191 (28.6%) | 5,872 (37.81%) |
| All other combinations | 1,445 (7.34%) | 245 (5.88%) | 1,200 (7.73%) |
| **Oral Cavity** | **12,890** | **2,867** | **10,023** |
| Surgery only | 1,766 (13.7%) | 844 (29.44%) | 922 (9.2%) |
| Chemotherapy only | 685 (5.31%) | 191 (6.66%) | 494 (4.93%) |
| Surgery + radiotherapy | 1,525 (11.83%) | 403 (14.06%) | 1,122 (11.19%) |
| Radiotherapy + chemotherapy | 3,073 (23.84%) | 415 (14.48%) | 2,658 (26.52%) |
| Surgery + chemotherapy + radiotherapy | 4,756 (36.9%) | 822 (28.67%) | 3,934 (39.25%) |
| All other combinations | 1,085 (8.42%) | 192 (6.70%) | 893 (8.91%) |
| **Oropharynx** | **2,489** | **390** | **2,099** |
| Surgery only | 42 (1.69%) | 17 (4.36%) | 25 (1.19%) |
| Chemotherapy only | 76 (3.05%) | 16 (4.1%) | 60 (2.86%) |
| Surgery + radiotherapy | 85 (3.42%) | 19 (4.87%) | 66 (3.14%) |
| Radiotherapy + chemotherapy | 1,478 (59.38%) | 232 (59.49%) | 1,246 (59.36%) |
| Surgery + chemotherapy + radiotherapy | 689 (27.68%) | 87 (22.31%) | 602 (28.68%) |
| All other combinations | 119 (4.78%) | 19 (4.87%) | 100 (4.76%) |
| **Hypopharynx** | **3,307** | **530** | **2,777** |
| Surgery only | 103 (3.11%) | 30 (5.66%) | 73 (2.63%) |
| Chemotherapy only | 80 (2.42%) | 20 (3.77%) | 60 (2.16%) |
| Surgery + radiotherapy | 216 (6.53%) | 51 (9.62%) | 165 (5.94%) |
| Radiotherapy + chemotherapy | 1,363 (41.22%) | 185 (34.91%) | 1,178 (42.42%) |
| Surgery + chemotherapy + radiotherapy | 1,354 (40.94%) | 220 (41.51%) | 1,134 (40.84%) |
| All other combinations | 191 (5.78%) | 24 (4.53%) | 167 (6.01%) |
| **Other** | **1,010** | **378** | **632** |
| Surgery only | 69 (6.83%) | 41 (10.85%) | 28 (4.43%) |
| Chemotherapy only | 187 (18.51%) | 119 (31.48%) | 68 (10.76%) |
| Surgery + radiotherapy | 95 (9.41%) | 36 (9.52%) | 59 (9.34%) |
| Radiotherapy + chemotherapy | 345 (34.16%) | 110 (29.1%) | 235 (37.18%) |
| Surgery + chemotherapy + radiotherapy | 264 (26.14%) | 62 (16.4%) | 202 (31.96%) |
| All other combinations | 50 (4.95%) | 10 (2.65%) | 40 (6.33%) |

**Supplementary eTable 9**. Treatment modalities by the tumor site and cancer stage in the targeted therapy group

| **Treatment modalities, N (%)** | **All** | **Stage III** | **Stage IVA-IVB** |
| --- | --- | --- | --- |
| **Overall** | **1,204** | **224** | **980** |
| Radiotherapy + targeted therapy | 230 (19.10%) | 69 (30.80%) | 161 (16.43%) |
| Radiotherapy + chemotherapy + targeted therapy | 569 (47.26%) | 81 (36.16%) | 488 (49.80%) |
| All other combinations | 405 (33.64%) | 74 (33.04%) | 331 (33.78%) |
| **Oral Cavity** | **252** | **42** | **210** |
| Radiotherapy + targeted therapy | 47 (18.65%) | 12 (28.57%) | 35 (16.67%) |
| Radiotherapy + chemotherapy + targeted therapy | 129 (51.19%) | 16 (38.10%) | 113 (53.81%) |
| All other combinations | 76 (30.16%) | 14 (33.33%) | 62 (29.52%) |
| **Oropharynx** | **305** | **45** | **260** |
| Radiotherapy + targeted therapy | 67 (21.97%) | 18 (40.00%) | 49 (18.85%) |
| Radiotherapy + chemotherapy + targeted therapy | 173 (56.72%) | 17 (37.78%) | 156 (60.00%) |
| All other combinations | 65 (21.31%) | 10 (22.22%) | 55 (21.15%) |
| **Hypopharynx** | **546** | **91** | **455** |
| Radiotherapy + targeted therapy | 88 (16.12%) | 23 (25.27%) | 65 (14.29%) |
| Radiotherapy + chemotherapy + targeted therapy | 235 (43.04%) | 32 (35.16%) | 203 (44.62%) |
| All other combinations | 223 (40.84%) | 36 (39.56%) | 187 (41.10%) |
| **Other** | **101** | **46** | **55** |
| Radiotherapy + targeted therapy | 28 (27.72%) | 16 (34.78%) | 12 (21.82%) |
| Radiotherapy + chemotherapy + targeted therapy | 32 (31.68%) | 16 (34.78%) | 16 (29.09%) |
| All other combinations | 41 (40.59%) | 14 (30.43%) | 27 (49.09%) |

**Supplementary eTable 10**. Treatment patterns of systemic therapy in the nontargeted therapy group (unit: times)

| **Treatment modalities** | **All** | **Stage III** | **Stage IVA-IVB** |
| --- | --- | --- | --- |
| **Induction therapy** | **12,699 (16.44%)** | **1,288 (11.27%)** | **11,411 (17.34%)** |
| Cisplatin + 5-FU + docetaxel | 3,763 (29.63%) | 169 (13.12%) | 3,594 (31.5%) |
| Cisplatin + 5-FU | 1,594 (12.55%) | 169 (13.12%) | 1,425 (12.49%) |
| Uracil-Tegafur | 1,379 (10.86%) | 193 (14.98%) | 1,186 (10.39%) |
| Cisplatin | 1,212 (9.54%) | 122 (9.47%) | 1,090 (9.55%) |
| Methotrexate + epirubicin | 878 (6.91%) | 159 (12.34%) | 719 (6.3%) |
| Other | 3,873 (30.5%) | 476 (36.96%) | 3,397 (29.77%) |
| **Definitive radiochemotherapy** | **21,598 (27.97%)** | **3,142 (27.48%)** | **18,456 (28.05%)** |
| Cisplatin | 11,179 (51.76%) | 1,811 (57.64%) | 9,368 (50.76%) |
| Cisplatin + 5-FU | 2,748 (12.72%) | 423 (13.46%) | 2,325 (12.6%) |
| Cisplatin + uracil-tegafur | 2,233 (10.34%) | 290 (9.23%) | 1,943 (10.53%) |
| Uracil-Tegafur | 1,796 (8.32%) | 241 (7.67%) | 1,555 (8.43%) |
| Cisplatin + 5-FU + docetaxel | 1,085 (5.02%) | 86 (2.74%) | 999 (5.41%) |
| All other combinations | 2,557 (11.84%) | 291 (9.26%) | 2,266 (12.28%) |
| **Postoperative radiochemotherapy** | **18,516 (23.98%)** | **3,166 (27.69%)** | **15,350 (23.33%)** |
| Cisplatin | 12,014 (64.88%) | 2,022 (63.87%) | 9,992 (65.09%) |
| Cisplatin + 5-FU | 2,281 (12.32%) | 395 (12.48%) | 1,886 (12.29%) |
| Cisplatin + uracil-tegafur | 1,579 (8.53%) | 269 (8.5%) | 1,310 (8.53%) |
| Uracil-Tegafur | 1,270 (6.86%) | 268 (8.46%) | 1,002 (6.53%) |
| Carboplatin | 427 (2.31%) | 76 (2.4%) | 351 (2.29%) |
| All other combinations | 945 (5.1%) | 136 (4.3%) | 809 (5.27%) |
| **Other** | **24,412 (31.61%)** | **3,836 (33.55%)** | **20,576 (31.27%)** |
| Cisplatin | 6,511 (26.67%) | 1,052 (27.42%) | 5,459 (26.53%) |
| Uracil-Tegafur | 4,705 (19.27%) | 866 (22.58%) | 3,839 (18.66%) |
| Cisplatin + 5-FU | 3,439 (14.09%) | 484 (12.62%) | 2,955 (14.36%) |
| Methotrexate | 1,659 (6.8%) | 264 (6.88%) | 1,395 (6.78%) |
| 5-FU + Methotrexate | 1,243 (5.09%) | 173 (4.51%) | 1,070 (5.2%) |
| All other combinations | 6,855 (28.08%) | 997 (25.99%) | 5,858 (28.47%) |

**Supplementary eTable 11**. Treatment patterns of systemic therapy in the targeted therapy group (unit: times)

| **Treatment modalities** | **All** | **Stage III** | **Stage IVA-IVB** |
| --- | --- | --- | --- |
| **Induction therapy** | **1,610 (17.22%)** | **96 (6.05%)** | **1,514 (19.51%)** |
| Cisplatin + 5-FU + docetaxel | 574 (35.65%) | 16 (16.67%) | 558 (36.86%) |
| Cisplatin | 163 (10.12%) | 9 (9.38%) | 154 (10.17%) |
| Uracil-Tegafur | 161 (10.00%) | 24 (25.00%) | 137 (9.05%) |
| 5-FU | 150 (9.32%) | 7 (7.29%) | 143 (9.45%) |
| Docetaxel | 142 (8.82%) | 8 (8.33%) | 134 (8.85%) |
| Other | 420 (26.09%) | 32 (33.33%) | 388 (25.63%) |
| **Definitive radiochemotherapy** | **3,896 (41.68%)** | **720 (45.4%)** | **3,176 (40.92%)** |
| Cetuximab | 2,871 (73.69%) | 573 (79.58%) | 2,298 (72.36%) |
| Cisplatin | 221 (5.67%) | 38 (5.28%) | 183 (5.76%) |
| Cetuximab + cisplatin | 136 (3.49%) | 9 (1.25%) | 127 (4.%) |
| Uracil-Tegafur | 113 (2.9%) | 28 (3.89%) | 85 (2.68%) |
| Cisplatin + 5-FU + docetaxel | 100 (2.57%) | 8 (1.11%) | 92 (2.9%) |
| All other combinations | 455 (11.68%) | 64 (8.89%) | 391 (12.31%) |
| **Postoperative radiochemotherapy** | **1,597 (17.08%)** | **311 (19.61%)** | **1,286 (16.57%)** |
| Cetuximab | 1,238 (77.52%) | 241 (77.49%) | 997 (77.53%) |
| Cetuximab + cisplatin | 66 (4.13%) | 21 (6.75%) | 45 (3.5%) |
| Cisplatin | 62 (3.88%) | 17 (5.47%) | 45 (3.5%) |
| Uracil-Tegafur | 55 (3.44%) | 10 (3.22%) | 45 (3.5%) |
| Cisplatin + 5-FU | 35 (2.19%) | 9 (2.89%) | 26 (2.02%) |
| All other combinations | 141 (8.83%) | 13 (4.18%) | 128 (9.95%) |
| **Other** | **2,245 (24.02%)** | **459 (28.94%)** | **1,786 (23.01%)** |
| Cetuximab | 1,587 (70.69%) | 331 (72.11%) | 1,256 (70.32%) |
| Uracil-Tegafur | 331 (14.74%) | 82 (17.86%) | 249 (13.94%) |
| Cisplatin + 5-FU | 64 (2.85%) | 8 (1.74%) | 56 (3.14%) |
| Cetuximab + Uracil-Tegafur | 45 (2.%) | 0 (%) | 45 (2.52%) |
| Cetuximab + cisplatin | 44 (1.96%) | 11 (2.4%) | 33 (1.85%) |
| All other combinations | 218 (9.71%) | 38 (8.28%) | 180 (10.08%) |

**Supplementary eTable 12**. All-cause mortality in the nontargeted versus targeted therapy group

| **Subgroup** | **Nontargeted therapy group** | | | |  | **Targeted therapy group** | | | | **MRR [95% CI]*** | ***P**** |
| --- | --- | --- | --- | --- | --- | --- | --- | --- | --- | --- | --- |
|  | **P-yrs** | **No. of**  **patients** | **No. of**  **deaths (%)** | **Mortality (100,000 p-yrs)** |  | **P-yrs** | **No. of**  **patients** | **No. of**  **deaths (%)** | **Mortality (100,000 p-yrs)** |  |  |
| **One-year mortality** | | | | | | | | | | | |
| **Overall** | 17,536.70 | 19,696 | 4,806 (24.4%) | 27,405.38 |  | 1,021.98 | 1,204 | 415 (34.47%) | 40,607.45 | 1.48 [1.34-1.64] | **<0.001** |
| **Age, years** |  |  |  |  |  |  |  |  |  |  |  |
| ≤ 44 | 3,035.64 | 3,305 | 675 (20.42%) | 22,235.84 |  | 41.19 | 47 | 15 (31.91%) | 36,416.61 | 1.64 [0.98-2.73] | 0.059 |
| 45-54 | 6,374.79 | 7,052 | 1,601 (22.7%) | 25,114.55 |  | 194.90 | 222 | 75 (33.78%) | 38,481.27 | 1.53 [1.22-1.93] | **<0.001** |
| 55-64 | 5,411.21 | 6,047 | 1,416 (23.42%) | 26,167.90 |  | 274.23 | 310 | 91 (29.35%) | 33,183.82 | 1.27 [1.03-1.57] | **0.028** |
| ≥ 65 | 2,715.06 | 3,292 | 1,114 (33.84%) | 41,030.40 |  | 511.66 | 625 | 234 (37.44%) | 45,733.49 | 1.11 [0.97-1.28] | 0.131 |
| **Sex** |  |  |  |  |  |  |  |  |  |  |  |
| Male | 16,283.03 | 18,289 | 4,483 (24.51%) | 27,531.73 |  | 970.03 | 1,144 | 397 (34.7%) | 40,926.57 | 1.49 [1.34-1.65] | **<0.001** |
| Female | 1,253.67 | 1,407 | 323 (22.96%) | 25,764.36 |  | 51.95 | 60 | 18 (30%) | 34,648.70 | 1.34 [0.84-2.16] | 0.221 |
| **Tumor stage** |  |  |  |  |  |  |  |  |  |  |  |
| III | 3,934.47 | 4,165 | 548 (13.16%) | 13,928.18 |  | 197.01 | 224 | 54 (24.11%) | 27,409.78 | 1.97 [1.49-2.60] | **<0.001** |
| IVA-IVB | 13,602.23 | 15,531 | 4,258 (27.42%) | 31,303.69 |  | 824.97 | 980 | 361 (36.84%) | 43,759.17 | 1.40 [1.26-1.56] | **<0.001** |
| **Tumor site** |  |  |  |  |  |  |  |  |  |  |  |
| Oral cavity | 11,486.51 | 12,890 | 3,157 (24.49%) | 27,484.41 |  | 206.56 | 252 | 97 (38.49%) | 46,959.72 | 1.71 [1.40-2.09] | **<0.001** |
| Oropharynx | 2,221.05 | 2,489 | 568 (22.82%) | 25,573.49 |  | 260.16 | 305 | 93 (30.49%) | 35,747.23 | 1.40 [1.12-1.74] | **0.003** |
| Hypopharynx | 2,899.02 | 3,307 | 906 (27.4%) | 31,251.94 |  | 468.35 | 546 | 198 (36.26%) | 42,276.08 | 1.35 [1.16-1.58] | **<0.001** |
| Others | 930.13 | 1,010 | 175 (17.33%) | 18,814.57 |  | 86.90 | 101 | 27 (26.73%) | 31,070.20 | 1.65 [1.10-2.48] | **0.015** |
| **Long-term mortality** | | | | | | | | | | | |
| **Overall** | 75,534.34 | 19,696 | 11,358 (57.67%) | 15,036.87 |  | 3,106.72 | 1,204 | 928 (77.08%) | 29,870.73 | 1.99 [1.86-2.12] | **<0.001** |
| **Age, year** |  |  |  |  |  |  |  |  |  |  |  |
| ≤ 44 | 13,923.70 | 3,305 | 1,800 (54.46%) | 12,927.60 |  | 127.97 | 47 | 38 (80.85%) | 29,694.46 | 2.30 [1.67-3.17] | **<0.001** |
| 45-54 | 28,016.75 | 7,052 | 3,950 (56.01%) | 14,098.71 |  | 593.07 | 222 | 169 (76.13%) | 28,495.79 | 2.02 [1.73-2.36] | **<0.001** |
| 55-64 | 23,444.35 | 6,047 | 3,335 (55.15%) | 14,225.18 |  | 919.03 | 310 | 217 (70%) | 23,611.85 | 1.66 [1.45-1.90] | **<0.001** |
| ≥ 65 | 10,149.54 | 3,292 | 2,273 (69.05%) | 22,395.10 |  | 1,466.65 | 625 | 504 (80.64%) | 34,364.03 | 1.53 [1.39-1.69] | **<0.001** |
| **Sex** |  |  |  |  |  |  |  |  |  |  |  |
| Male | 69,723.98 | 18,289 | 10,671 (58.35%) | 15,304.63 |  | 2,917.53 | 1,144 | 892 (77.97%) | 30,573.81 | 2.00 [1.87-2.14] | **<0.001** |
| Female | 5,810.35 | 1,407 | 687 (48.83%) | 11,823.73 |  | 189.19 | 60 | 36 (60%) | 19,028.49 | 1.61 [1.15-2.25] | **0.005** |
| **Tumor stage** |  |  |  |  |  |  |  |  |  |  |  |
| III | 20,156.45 | 4,165 | 1,827 (43.87%) | 9,064.10 |  | 713.47 | 224 | 151 (67.41%) | 21,164.17 | 2.33 [1.98-2.76] | **<0.001** |
| IVA-IVB | 55,377.89 | 15,531 | 9,531 (61.37%) | 17,210.84 |  | 2,393.24 | 980 | 777 (79.29%) | 32,466.45 | 1.89 [1.75-2.03] | **<0.001** |
| **Tumor site** |  |  |  |  |  |  |  |  |  |  |  |
| Oral cavity | 51,372.76 | 12,890 | 7,159 (55.54%) | 13,935.40 |  | 559.44 | 252 | 207 (82.14%) | 37,001.29 | 2.66 [2.31-3.05] | **<0.001** |
| Oropharynx | 9,635.70 | 2,489 | 1,382 (55.52%) | 14,342.50 |  | 898.98 | 305 | 213 (69.84%) | 23,693.52 | 1.65 [1.43-1.91] | **<0.001** |
| Hypopharynx | 10,759.09 | 3,307 | 2,316 (70.03%) | 21,525.98 |  | 1,385.63 | 546 | 441 (80.77%) | 31,826.68 | 1.48 [1.34-1.64] | **<0.001** |
| Others | 3,766.78 | 1,010 | 501 (49.6%) | 13,300.48 |  | 262.66 | 101 | 67 (66.34%) | 25,508.26 | 1.92 [1.49-2.47] | **<0.001** |

p-yrs= person-years; MRR= mortality rate ratio; CI= confidence interval.

*The MRR and p-value were compared targeted therapy group to the targeted one.

**Supplementary eTable 13.** Cancer-specific mortality in the nontargeted versus targeted therapy group

| **Subgroup** | **Nontargeted therapy group** | | | |  | **Targeted-therapy group** | | | | **MRR [95% CI]** | ***P*** |
| --- | --- | --- | --- | --- | --- | --- | --- | --- | --- | --- | --- |
|  | **P-yrs** | **No. of**  **patients** | **No. of**  **deaths (%)** | **Mortality (100,000 p-yrs)** |  | **P-yrs** | **No. of**  **patients** | **No. of**  **deaths (%)** | **Mortality (100,000 p-yrs)** |  |  |
| **One-year mortality** | | | | | | | | | | | |
| **Overall** | 17,536.70 | 19,696 | 4,097 (20.8%) | 23,362.43 |  | 1,021.98 | 1,204 | 347 (28.82%) | 23,362.43 | 1.45 [1.30-1.62] | **<0.001** |
| **Age, year** |  |  |  |  |  |  |  |  |  |  |  |
| ≤ 44 | 3,035.64 | 3,305 | 591 (17.88%) | 19,468.71 |  | 41.19 | 47 | 15 (31.91%) | 19,468.71 | 1.87 [1.12-3.12] | **0.017** |
| 45-54 | 6,374.79 | 7,052 | 1,373 (19.47%) | 21,537.96 |  | 194.90 | 222 | 71 (31.98%) | 21,537.96 | 1.69 [1.33-2.15] | **<0.001** |
| 55-64 | 5,411.21 | 6,047 | 1,210 (20.01%) | 22,360.99 |  | 274.23 | 310 | 73 (23.55%) | 22,360.99 | 1.19 [0.94-1.51] | 0.148 |
| ≥ 65 | 2,715.06 | 3,292 | 923 (28.04%) | 33,995.57 |  | 511.66 | 625 | 188 (30.08%) | 33,995.57 | 1.08 [0.92-1.26] | 0.331 |
| **Sex** |  |  |  |  |  |  |  |  |  |  |  |
| Male | 16,283.03 | 18,289 | 3,815 (20.86%) | 23,429.30 |  | 970.03 | 1,144 | 329 (28.76%) | 23,429.30 | 1.45 [1.29-1.62] | **<0.001** |
| Female | 1,253.67 | 1,407 | 282 (20.04%) | 22,493.96 |  | 51.95 | 60 | 18 (30%) | 22,493.96 | 1.54 [0.96-2.48] | 0.076 |
| **Tumor stage** |  |  |  |  |  |  |  |  |  |  |  |
| III | 3,934.47 | 4,165 | 432 (10.37%) | 10,979.88 |  | 197.01 | 224 | 39 (17.41%) | 10,979.88 | 1.80 [1.30-2.50] | **<0.001** |
| IVA-IVB | 13,602.23 | 15,531 | 3,665 (23.6%) | 26,944.11 |  | 824.97 | 980 | 308 (31.43%) | 26,944.11 | 1.39 [1.23-1.56] | **<0.001** |
| **Tumor site** |  |  |  |  |  |  |  |  |  |  |  |
| Oral cavity | 11,486.51 | 12,890 | 2,766 (21.46%) | 24,080.42 |  | 206.56 | 252 | 89 (35.32%) | 24,080.42 | 1.79 [1.45-2.21] | **<0.001** |
| Oropharynx | 2,221.05 | 2,489 | 458 (18.4%) | 20,620.88 |  | 260.16 | 305 | 71 (23.28%) | 20,620.88 | 1.32 [1.03-1.70] | **0.028** |
| Hypopharynx | 2,899.02 | 3,307 | 730 (22.07%) | 25,180.92 |  | 468.35 | 546 | 167 (30.59%) | 25,180.92 | 1.42 [1.20-1.68] | **<0.001** |
| Others | 930.13 | 1,010 | 143 (14.16%) | 15,374.20 |  | 86.90 | 101 | 20 (19.8%) | 15,374.20 | 1.50 [0.94-2.39] | 0.091 |
| **Long-term mortality** | | | | | | | | | | | |
| **Overall** | 75,534.34 | 19,696 | 8,906 (45.22%) | 11,790.66 |  | 3,106.72 | 1,204 | 706 (58.64%) | 22,724.93 | 1.93 [1.79-2.08] | **<0.001** |
| **Age, year** |  |  |  |  |  |  |  |  |  |  |  |
| ≤ 44 | 13,923.70 | 3,305 | 1,488 (45.02%) | 10,686.81 |  | 127.97 | 47 | 31 (65.96%) | 24,224.43 | 2.27 [1.59-3.23] | **<0.001** |
| 45-54 | 28,016.75 | 7,052 | 3,176 (45.04%) | 11,336.08 |  | 593.07 | 222 | 140 (63.06%) | 23,605.98 | 2.08 [1.76-2.47] | **<0.001** |
| 55-64 | 23,444.35 | 6,047 | 2,590 (42.83%) | 11,047.44 |  | 919.03 | 310 | 164 (52.9%) | 17,844.90 | 1.62 [1.38-1.89] | **<0.001** |
| ≥ 65 | 10,149.54 | 3,292 | 1,652 (50.18%) | 16,276.60 |  | 1,466.65 | 625 | 371 (59.36%) | 25,295.74 | 1.55 [1.39-1.74] | **<0.001** |
| **Sex** |  |  |  |  |  |  |  |  |  |  |  |
| Male | 69,723.98 | 18,289 | 8,362 (45.72%) | 11,993.00 |  | 2,917.53 | 1,144 | 673 (58.83%) | 23,067.46 | 1.92 [1.78-2.08] | **<0.001** |
| Female | 5,810.35 | 1,407 | 544 (38.66%) | 9,362.60 |  | 189.19 | 60 | 33 (55%) | 17,442.78 | 1.86 [1.31-2.65] | **0.001** |
| **Tumor stage** |  |  |  |  |  |  |  |  |  |  |  |
| III | 20,156.45 | 4,165 | 1,294 (31.07%) | 6,419.78 |  | 713.47 | 224 | 95 (42.41%) | 13,315.21 | 2.07 [1.68-2.55] | **<0.001** |
| IVA-IVB | 55,377.89 | 15,531 | 7,612 (49.01%) | 13,745.56 |  | 2,393.24 | 980 | 611 (62.35%) | 25,530.24 | 1.86 [1.71-2.02] | **<0.001** |
| **Tumor site** |  |  |  |  |  |  |  |  |  |  |  |
| Oral cavity | 51,372.76 | 12,890 | 5,814 (45.1%) | 11,317.28 |  | 559.44 | 252 | 168 (66.67%) | 30,030.03 | 2.65 [2.28-3.09] | **<0.001** |
| Oropharynx | 9,635.70 | 2,489 | 976 (39.21%) | 10,129.00 |  | 898.98 | 305 | 150 (49.18%) | 16,685.58 | 1.65 [1.39-1.96] | **<0.001** |
| Hypopharynx | 10,759.09 | 3,307 | 1,746 (52.8%) | 16,228.14 |  | 1,385.63 | 546 | 343 (62.82%) | 24,754.08 | 1.53 [1.36-1.71] | **<0.001** |
| Others | 3,766.78 | 1,010 | 370 (36.63%) | 9,822.71 |  | 262.66 | 101 | 45 (44.55%) | 17,132.41 | 1.74 [1.28-2.38] | **<0.001** |

p-yrs= person-years; MRR= mortality rate ratio; CI= confidence interval.

*The MRR and p-value were compared targeted therapy group to the targeted one.

**Supplementary eTable 14.** Summarized results from Cox proportional hazard regression models for the association between treatment modalities with targeted and nontargeted therapy and all-cause, cancer-specific mortality among locally advanced head and neck cancer patients (overall, by tumor stages, and cancer sites)

| **Subgroups** | **Treatment modality** | **All-cause mortality** | | | | **Cancer-specific mortality** | | | |
| --- | --- | --- | --- | --- | --- | --- | --- | --- | --- |
|  |  | **One-year survival** | | **Long-term** | | **One-year survival** | | **Long-term** | |
|  |  | **HR (95% CI)** | ***P*** | **HR (95% CI)** | ***P*** | **HR (95% CI)** | **P-value** | **HR (95% CI)** | ***P*** |
| **Overall** | Surgery only (Ref.) |  |  |  |  |  |  |  |  |
|  | Surgery + radiotherapy | 4.44 (3.29-6.00) | <0.001 | 1.32 (1.18-1.47) | <0.001 | 5.28 (3.75-7.43) | <0.001 | 1.52 (1.34-1.73) | <0.001 |
|  | Surgery + radiotherapy + chemotherapy | 9.38 (7.27-12.10) | <0.001 | 1.95 (1.78-2.12) | <0.001 | 11.32 (8.43-15.19) | <0.001 | 2.37 (2.13-2.64) | <0.001 |
|  | Surgery + radiotherapy + chemotherapy + targeted therapy | 12.33 (8.07-18.83) | <0.001 | 2.48 (2.09-2.94) | <0.001 | 14.20 (8.77-22.97) | <0.001 | 3.14 (2.59-3.82) | <0.001 |
|  | Radiotherapy only | 6.05 (4.52-8.10) | <0.001 | 2.50 (2.23-2.80) | <0.001 | 6.64 (4.76-9.26) | <0.001 | 2.74 (2.39-3.15) | <0.001 |
|  | Radiotherapy + chemotherapy | 9.98 (7.75-12.86) | <0.001 | 2.54 (2.33-2.78) | <0.001 | 12.42 (9.28-16.64) | <0.001 | 3.19 (2.87-3.55) | <0.001 |
|  | Radiotherapy + targeted therapy | 10.84 (7.21-16.30) | <0.001 | 3.20 (2.69-3.80) | <0.001 | 11.80 (7.41-18.79) | <0.001 | 3.77 (3.07-4.62) | <0.001 |
|  | Radiotherapy + chemotherapy + targeted therapy | 14.83 (10.66-20.63) | <0.001 | 2.98 (2.62-3.38) | <0.001 | 19.85 (13.72-28.71) | <0.001 | 3.95 (3.40-4.58) | <0.001 |
|  | All other combinations | 7.48 (5.74-9.75) | <0.001 | 3.12 (2.82-3.45) | <0.001 | 8.99 (6.63-12.19) | <0.001 | 3.78 (3.35-4.26) | <0.001 |
| **Tumor stage** |  |  |  |  |  |  |  |  |  |
| III | Surgery only (Ref.) |  |  |  |  |  |  |  |  |
|  | Surgery + radiotherapy | 8.04 (4.37-14.77) | <0.001 | 2.16 (1.75-2.67) | <0.001 | 10.70 (5.18-22.11) | <0.001 | 2.63 (2.03-3.40) | <0.001 |
|  | Surgery + radiotherapy + chemotherapy | 13.44 (7.84-23.03) | <0.001 | 2.81 (2.37-3.35) | <0.001 | 18.45 (9.72-35.04) | <0.001 | 3.73 (3.01-4.61) | <0.001 |
|  | Surgery + radiotherapy + chemotherapy + targeted therapy | 24.16 (8.87-65.86) | <0.001 | 3.87 (2.52-5.94) | <0.001 | 28.33 (9.08-88.42) | <0.001 | 5.13 (3.14-8.38) | <0.001 |
|  | Radiotherapy only | 6.64 (3.61-12.23) | <0.001 | 2.88 (2.34-3.55) | <0.001 | 5.88 (2.83-12.21) | <0.001 | 3.25 (2.49-4.24) | <0.001 |
|  | Radiotherapy + chemotherapy | 11.94 (6.90-20.67) | <0.001 | 2.86 (2.38-3.44) | <0.001 | 14.15 (7.34-27.27) | <0.001 | 3.71 (2.96-4.65) | <0.001 |
|  | Radiotherapy + targeted therapy | 9.75 (4.17-22.80) | <0.001 | 3.43 (2.45-4.81) | <0.001 | 6.85 (2.25-20.83) | 0.001 | 3.53 (2.24-5.57) | <0.001 |
|  | Radiotherapy + chemotherapy + targeted therapy | 9.61 (4.06-22.77) | <0.001 | 3.04 (2.19-4.22) | <0.001 | 13.14 (4.87-35.44) | <0.001 | 4.17 (2.82-6.18) | <0.001 |
|  | All other combinations | 13.12 (7.21-23.85) | <0.001 | 3.34 (2.68-4.15) | <0.001 | 14.76 (7.17-30.38) | <0.001 | 3.94 (3.01-5.15) | <0.001 |
| IVA-B | Surgery only (Ref.) |  |  |  |  |  |  |  |  |
|  | Surgery + radiotherapy | 3.54 (2.51-4.99) | <0.001 | 1.14 (1.01-1.30) | 0.042 | 4.18 (2.84-6.16) | <0.001 | 1.32 (1.13-1.53) | <0.001 |
|  | Surgery + radiotherapy + chemotherapy | 7.99 (5.99-10.67) | <0.001 | 1.77 (1.59-1.97) | <0.001 | 9.70 (6.96-13.52) | <0.001 | 2.15 (1.90-2.44) | <0.001 |
|  | Surgery + radiotherapy + chemotherapy + targeted therapy | 10.00 (6.26-15.97) | <0.001 | 2.23 (1.84-2.69) | <0.001 | 11.73 (6.90-19.95) | <0.001 | 2.82 (2.28-3.51) | <0.001 |
|  | Radiotherapy only | 5.49 (3.94-7.66) | <0.001 | 2.32 (2.03-2.66) | <0.001 | 6.47 (4.44-9.41) | <0.001 | 2.56 (2.18-3.00) | <0.001 |
|  | Radiotherapy + chemotherapy | 8.67 (6.51-11.55) | <0.001 | 2.39 (2.15-2.66) | <0.001 | 11.06 (7.96-15.36) | <0.001 | 3.00 (2.64-3.40) | <0.001 |
|  | Radiotherapy + targeted therapy | 10.42 (6.56-16.54) | <0.001 | 3.02 (2.47-3.69) | <0.001 | 12.39 (7.42-20.69) | <0.001 | 3.69 (2.93-4.64) | <0.001 |
|  | Radiotherapy + chemotherapy + targeted therapy | 13.99 (9.73-20.12) | <0.001 | 2.87 (2.49-3.32) | <0.001 | 18.84 (12.56-28.25) | <0.001 | 3.80 (3.21-4.49) | <0.001 |
|  | All other combinations | 6.16 (4.57-8.31) | <0.001 | 2.96 (2.63-3.33) | <0.001 | 7.73 (5.50-10.87) | <0.001 | 3.61 (3.14-4.16) | <0.001 |
| **Tumor sites** |  |  |  |  |  |  |  |  |  |
| Oral cavity | Surgery only (Ref.) |  |  |  |  |  |  |  |  |
|  | Surgery + radiotherapy | 6.17 (4.28-8.88) | <0.001 | 1.42 (1.25-1.61) | <0.001 | 7.70 (5.07-11.70) | <0.001 | 1.66 (1.43-1.93) | <0.001 |
|  | Surgery + radiotherapy + chemotherapy | 14.20 (10.41-19.37) | <0.001 | 2.39 (2.16-2.65) | <0.001 | 18.16 (12.64-26.08) | <0.001 | 2.92 (2.59-3.30) | <0.001 |
|  | Surgery + radiotherapy + chemotherapy + targeted therapy | 12.87 (5.29-31.31) | <0.001 | 3.14 (2.22-4.42) | <0.001 | 19.75 (7.73-50.43) | <0.001 | 4.05 (2.79-5.87) | <0.001 |
|  | Radiotherapy only | 8.74 (6.16-12.41) | <0.001 | 2.89 (2.53-3.30) | <0.001 | 10.64 (7.11-15.91) | <0.001 | 3.24 (2.77-3.80) | <0.001 |
|  | Radiotherapy + chemotherapy | 14.94 (10.97-20.33) | <0.001 | 3.31 (2.99-3.67) | <0.001 | 19.32 (13.48-27.69) | <0.001 | 4.18 (3.70-4.73) | <0.001 |
|  | Radiotherapy + targeted therapy | 13.94 (6.61-29.38) | <0.001 | 3.71 (2.65-5.18) | <0.001 | 18.14 (8.26-39.84) | <0.001 | 5.22 (3.66-7.44) | <0.001 |
|  | Radiotherapy + chemotherapy + targeted therapy | 19.64 (11.69-33.00) | <0.001 | 4.17 (3.37-5.16) | <0.001 | 27.63 (15.73-48.53) | <0.001 | 5.15 (4.04-6.56) | <0.001 |
|  | All other combinations | 9.65 (6.99-13.33) | <0.001 | 3.35 (2.98-3.77) | <0.001 | 12.12 (8.33-17.63) | <0.001 | 4.07 (3.54-4.67) | <0.001 |
| Oropharynx | Surgery only (Ref.) |  |  |  |  |  |  |  |  |
|  | Surgery + radiotherapy | 1.17 (0.32-4.19) | 0.813 | 0.89 (0.54-1.48) | 0.651 | 1.09 (0.23-5.12) | 0.916 | 0.89 (0.47-1.71) | 0.736 |
|  | Surgery + radiotherapy + chemotherapy | 1.82 (0.62-5.34) | 0.277 | 0.71 (0.46-1.10) | 0.124 | 1.86 (0.52-6.62) | 0.336 | 0.81 (0.47-1.40) | 0.446 |
|  | Surgery + radiotherapy + chemotherapy + targeted therapy | 2.30 (0.59-8.99) | 0.230 | 1.08 (0.61-1.90) | 0.801 | 2.07 (0.42-10.16) | 0.370 | 1.31 (0.65-2.65) | 0.455 |
|  | Radiotherapy only | 1.81 (0.55-5.96) | 0.330 | 1.66 (1.01-2.71) | 0.044 | 1.10 (0.26-4.70) | 0.900 | 1.62 (0.87-3.04) | 0.130 |
|  | Radiotherapy + chemotherapy | 1.95 (0.69-5.55) | 0.209 | 0.98 (0.64-1.48) | 0.907 | 2.13 (0.62-7.31) | 0.230 | 1.12 (0.66-1.92) | 0.672 |
|  | Radiotherapy + targeted therapy | 1.70 (0.47-6.05) | 0.416 | 1.39 (0.83-2.32) | 0.207 | 1.21 (0.26-5.68) | 0.810 | 1.37 (0.71-2.62) | 0.349 |
|  | Radiotherapy + chemotherapy + targeted therapy | 3.10 (1.01-9.48) | 0.048 | 1.35 (0.86-2.13) | 0.192 | 3.62 (0.97-13.44) | 0.055 | 1.71 (0.97-3.03) | 0.065 |
|  | All other combinations | 2.16 (0.71-6.53) | 0.173 | 1.81 (1.14-2.88) | 0.012 | 1.97 (0.53-7.28) | 0.312 | 1.95 (1.08-3.53) | 0.027 |
| Hypopharynx | Surgery only (Ref.) |  |  |  |  |  |  |  |  |
|  | Surgery + radiotherapy | 0.93 (0.48-1.81) | 0.840 | 0.59 (0.44-0.79) | <0.001 | 0.93 (0.45-1.95) | 0.857 | 0.64 (0.46-0.91) | 0.012 |
|  | Surgery + radiotherapy + chemotherapy | 1.29 (0.75-2.22) | 0.363 | 0.63 (0.49-0.80) | <0.001 | 1.30 (0.71-2.39) | 0.396 | 0.68 (0.51-0.91) | 0.009 |
|  | Surgery + radiotherapy + chemotherapy + targeted therapy | 1.97 (1.01-3.85) | 0.048 | 0.84 (0.62-1.13) | 0.254 | 1.91 (0.90-4.04) | 0.092 | 0.93 (0.66-1.33) | 0.703 |
|  | Radiotherapy only | 1.46 (0.71-2.97) | 0.301 | 1.15 (0.82-1.60) | 0.426 | 1.26 (0.58-2.78) | 0.559 | 1.18 (0.80-1.74) | 0.399 |
|  | Radiotherapy + chemotherapy | 1.41 (0.83-2.40) | 0.208 | 0.78 (0.61-0.99) | 0.039 | 1.46 (0.81-2.65) | 0.209 | 0.87 (0.65-1.16) | 0.337 |
|  | Radiotherapy + targeted therapy | 2.07 (1.03-4.16) | 0.040 | 1.21 (0.87-1.67) | 0.252 | 2.12 (0.98-4.58) | 0.057 | 1.21 (0.83-1.76) | 0.328 |
|  | Radiotherapy + chemotherapy + targeted therapy | 2.64 (1.45-4.83) | 0.002 | 0.99 (0.75-1.30) | 0.942 | 2.88 (1.48-5.57) | 0.002 | 1.18 (0.86-1.61) | 0.317 |
|  | All other combinations | 1.42 (0.80-2.51) | 0.229 | 1.29 (0.98-1.68) | 0.068 | 1.62 (0.86-3.05) | 0.137 | 1.49 (1.08-2.04) | 0.014 |
| Others | Surgery only (Ref.) |  |  |  |  |  |  |  |  |
|  | Surgery + radiotherapy | 1.71 (0.44-6.70) | 0.443 | 0.90 (0.56-1.44) | 0.657 | 2.02 (0.41-9.90) | 0.385 | 1.12 (0.62-2.02) | 0.714 |
|  | Surgery + radiotherapy + chemotherapy | 1.48 (0.42-5.14) | 0.540 | 1.11 (0.74-1.66) | 0.623 | 1.43 (0.33-6.25) | 0.633 | 1.54 (0.92-2.59) | 0.101 |
|  | Surgery + radiotherapy + chemotherapy + targeted therapy | 4.72 (0.91-24.38) | 0.064 | 1.34 (0.69-2.59) | 0.386 | 3.42 (0.46-25.41) | 0.229 | 1.64 (0.75-3.57) | 0.216 |
|  | Radiotherapy only | 0.60 (0.16-2.29) | 0.453 | 0.94 (0.62-1.43) | 0.763 | 0.56 (0.11-2.79) | 0.483 | 1.02 (0.58-1.78) | 0.944 |
|  | Radiotherapy + chemotherapy | 1.48 (0.44-5.04) | 0.530 | 1.13 (0.76-1.67) | 0.557 | 1.68 (0.40-7.17) | 0.480 | 1.35 (0.81-2.26) | 0.253 |
|  | Radiotherapy + targeted therapy | 2.62 (0.57-12.05) | 0.217 | 1.75 (0.97-3.17) | 0.062 | 1.87 (0.28-12.50) | 0.519 | 1.66 (0.75-3.69) | 0.214 |
|  | Radiotherapy + chemotherapy + targeted therapy | 0.70 (0.12-4.22) | 0.696 | 1.44 (0.82-2.55) | 0.206 | 1.02 (0.14-7.14) | 0.987 | 1.77 (0.87-3.59) | 0.116 |
|  | All other combinations | 3.34 (0.94-11.87) | 0.063 | 2.45 (1.57-3.82) | <0.001 | 3.92 (0.88-17.47) | 0.073 | 3.31 (1.88-5.82) | <0.001 |
